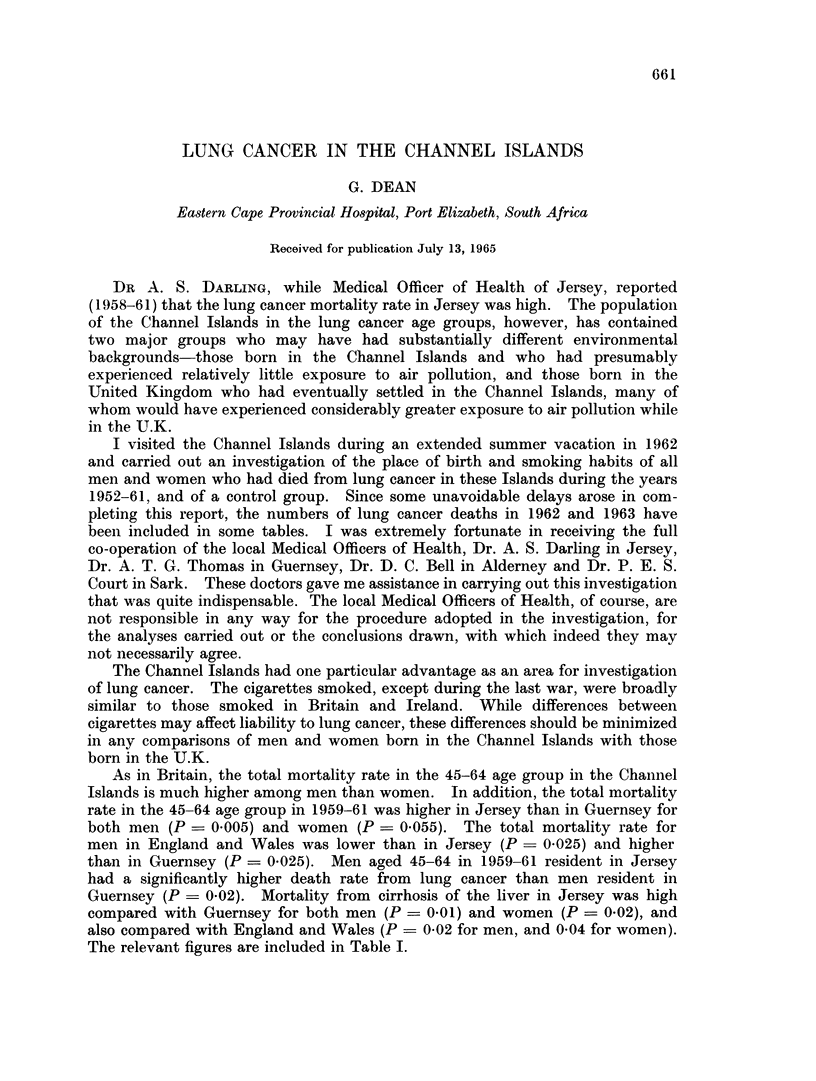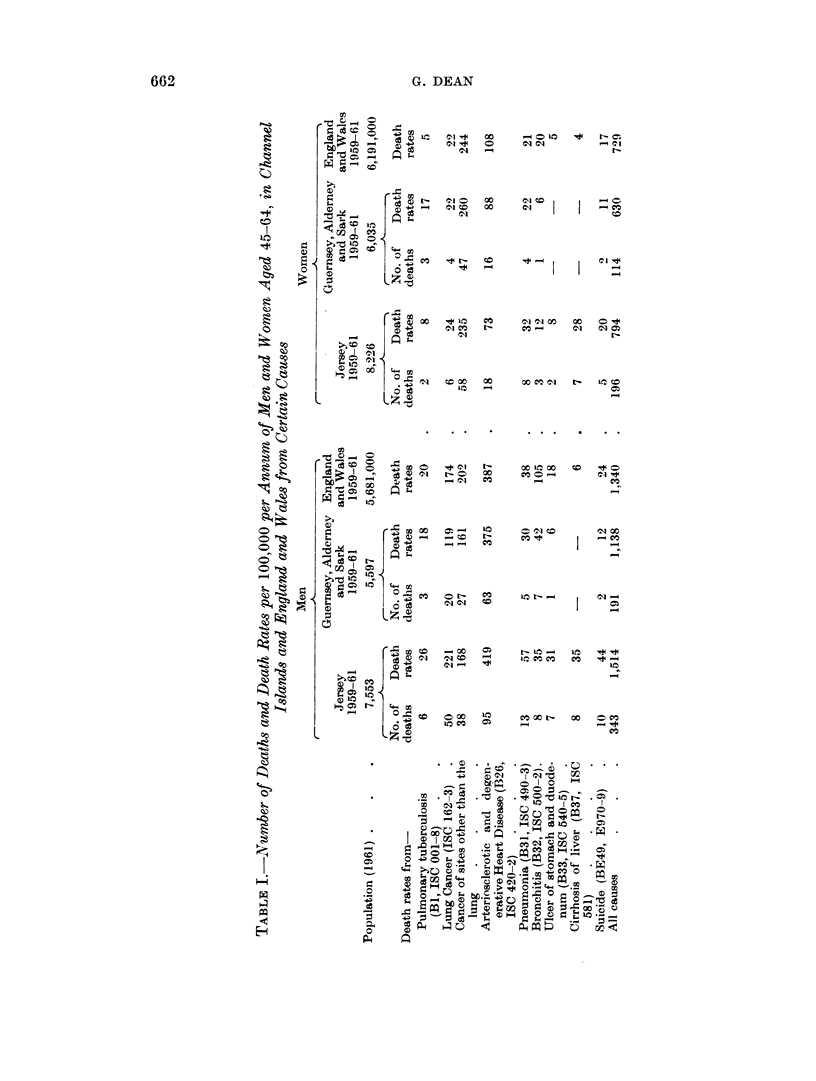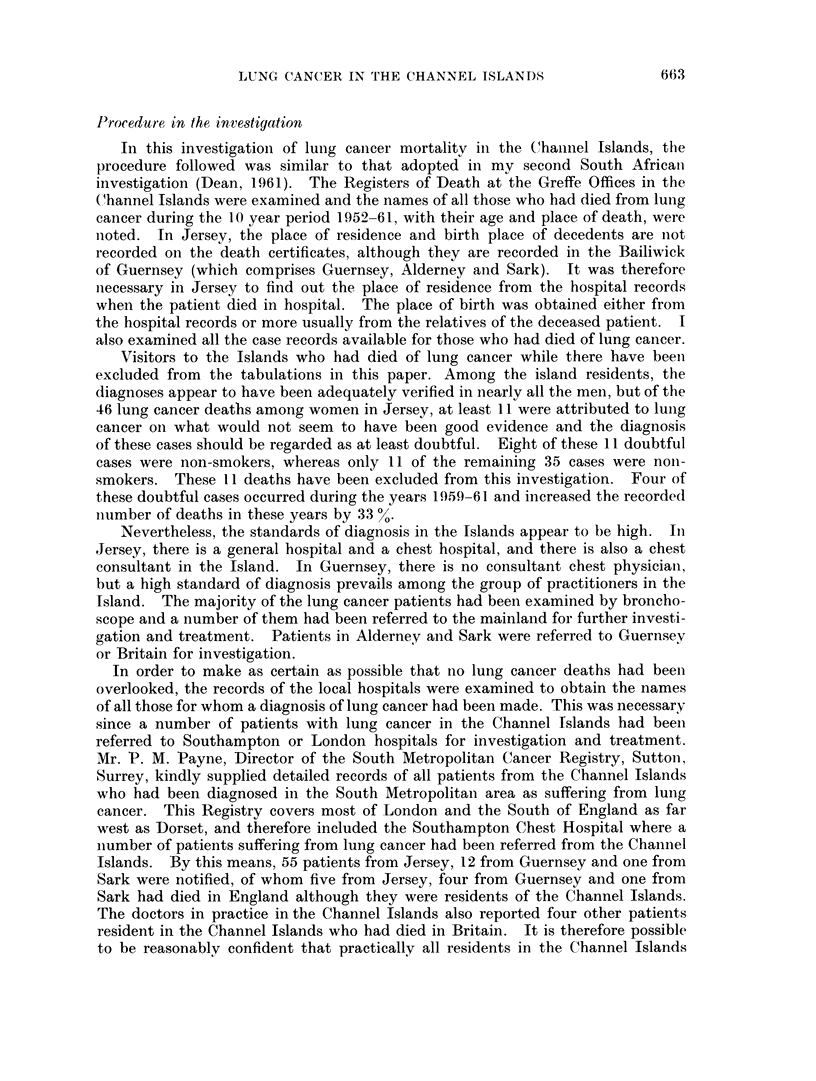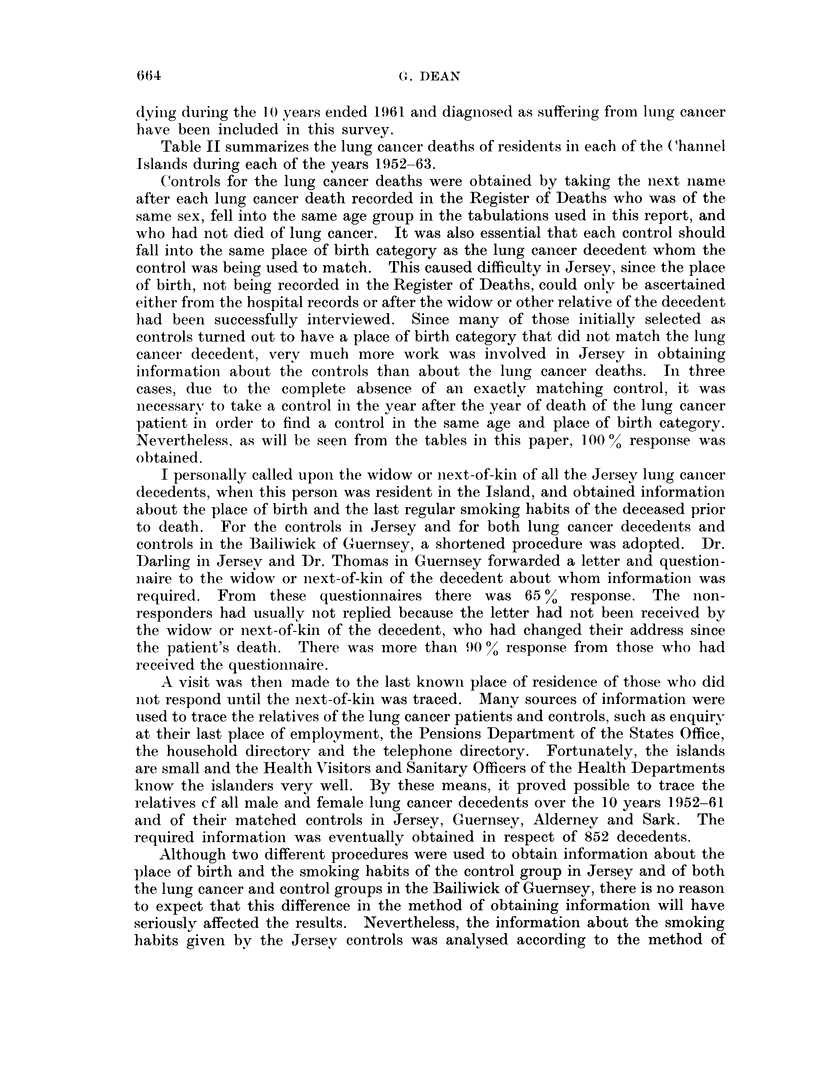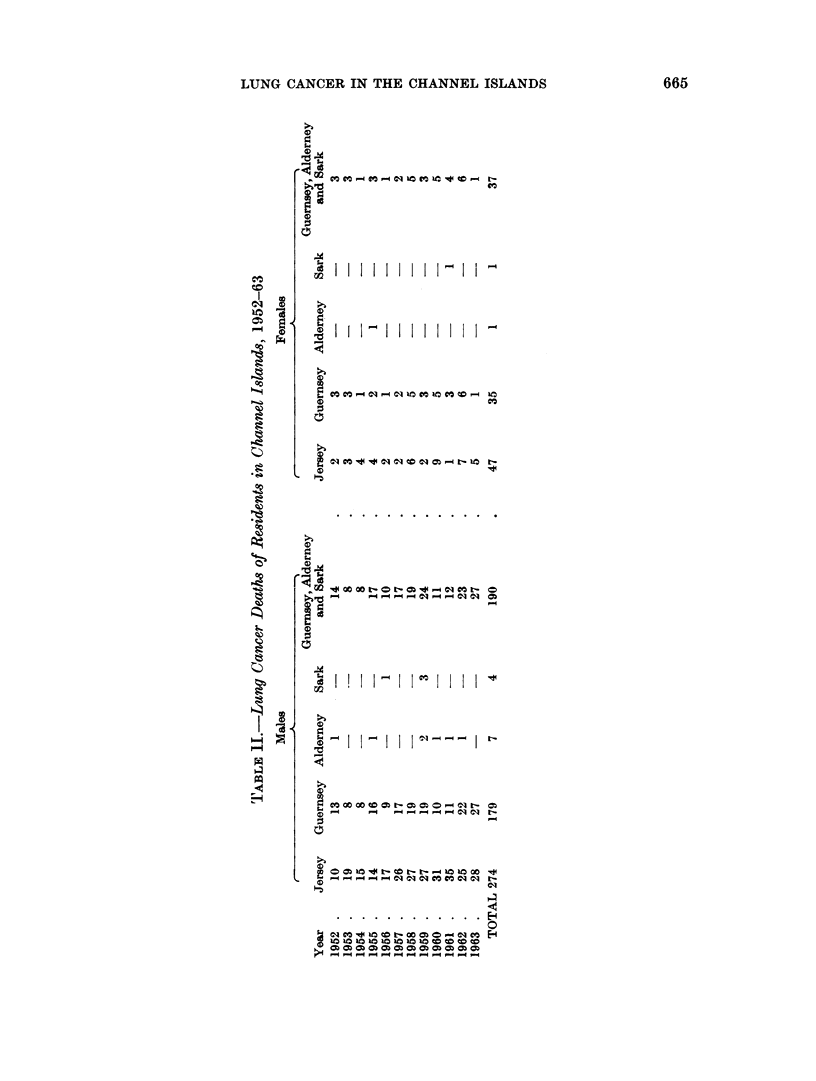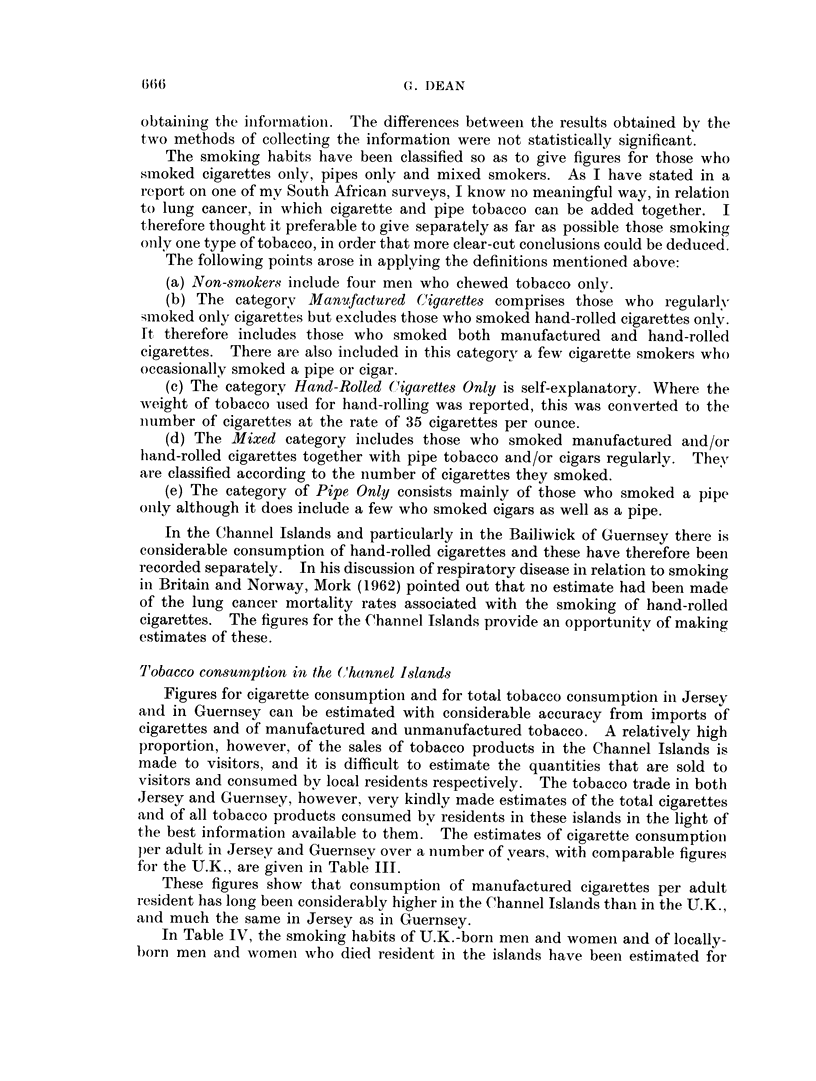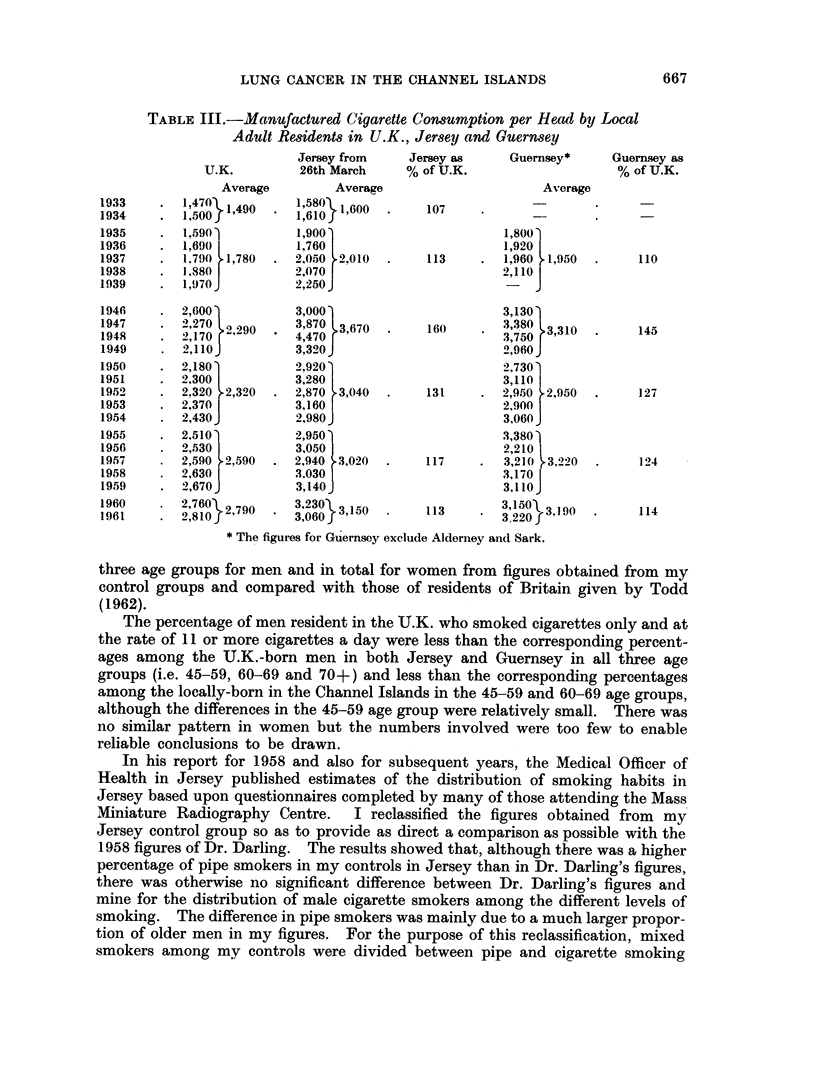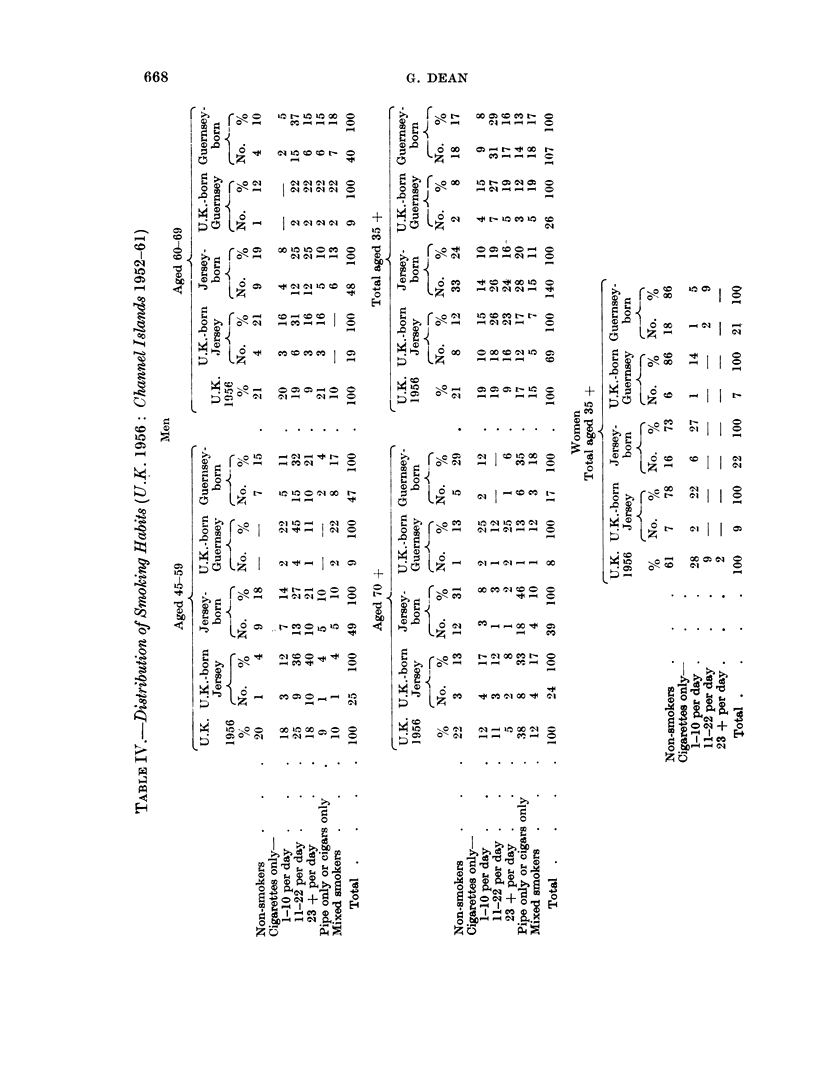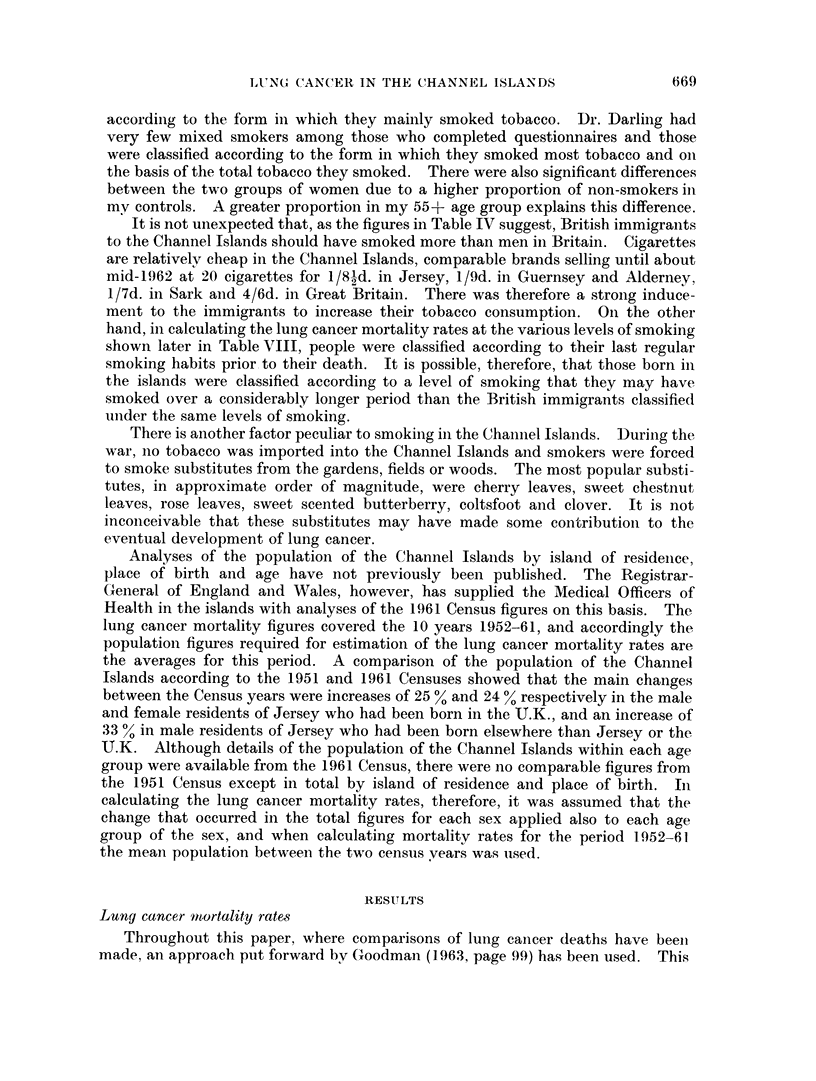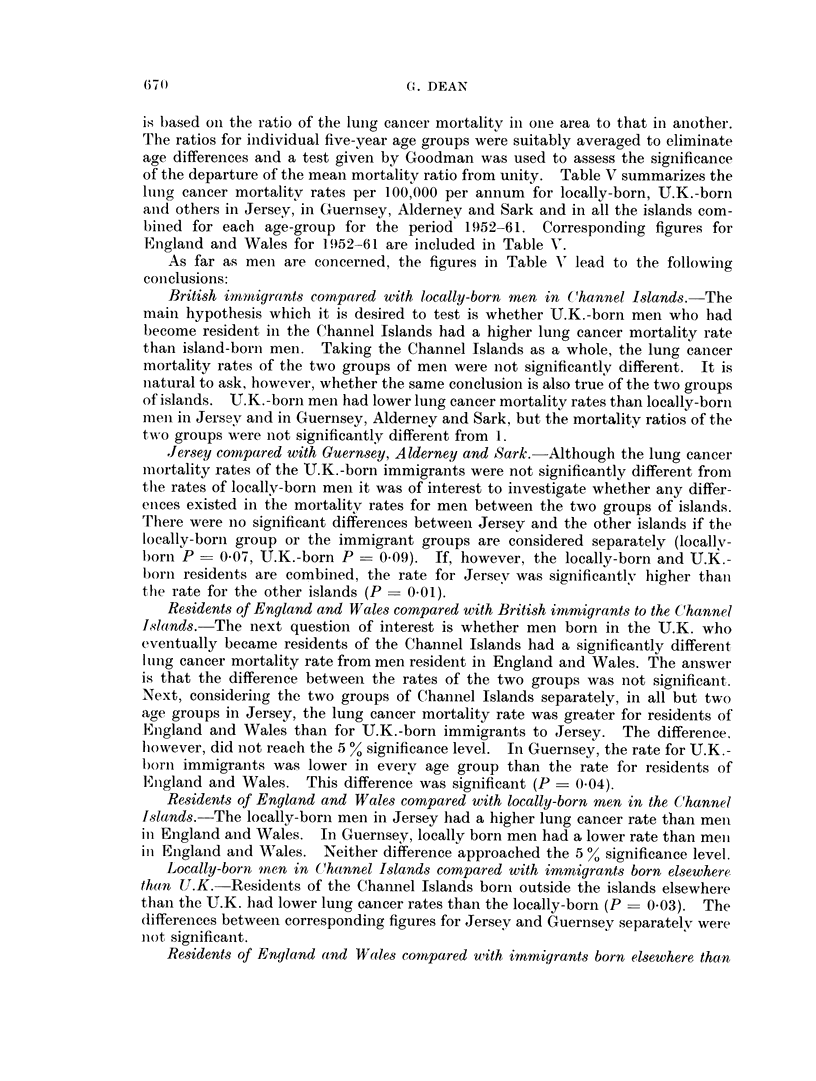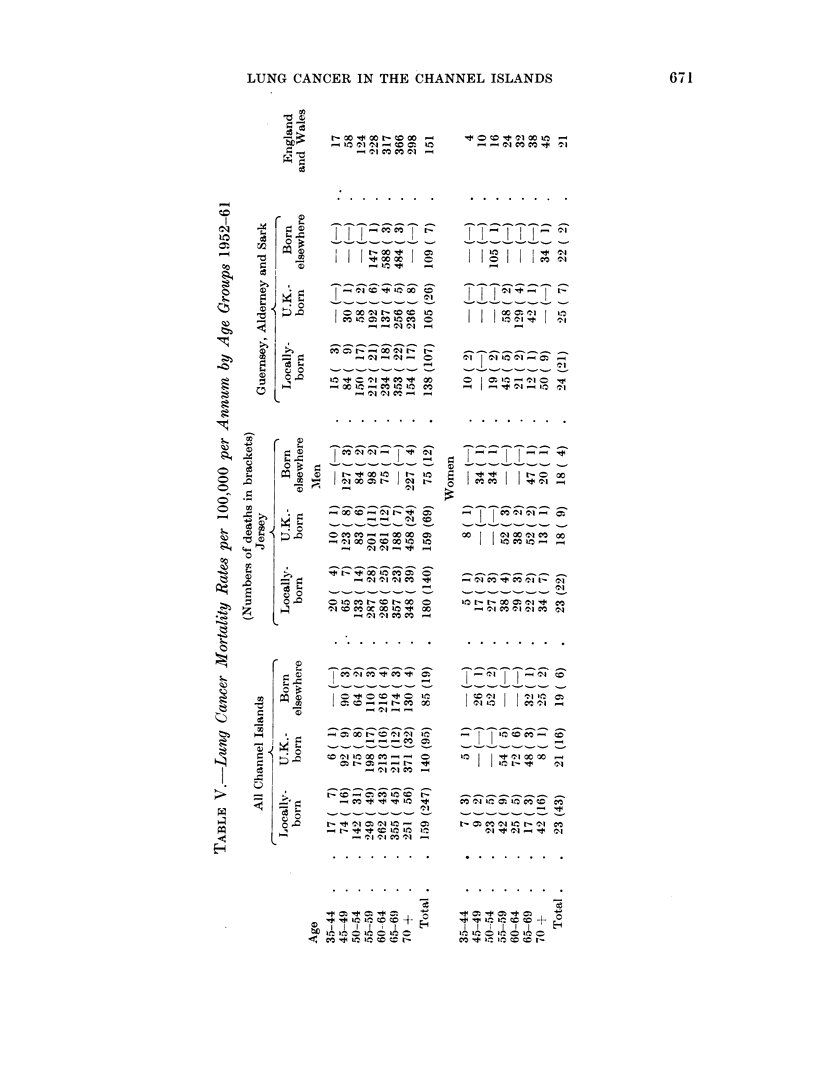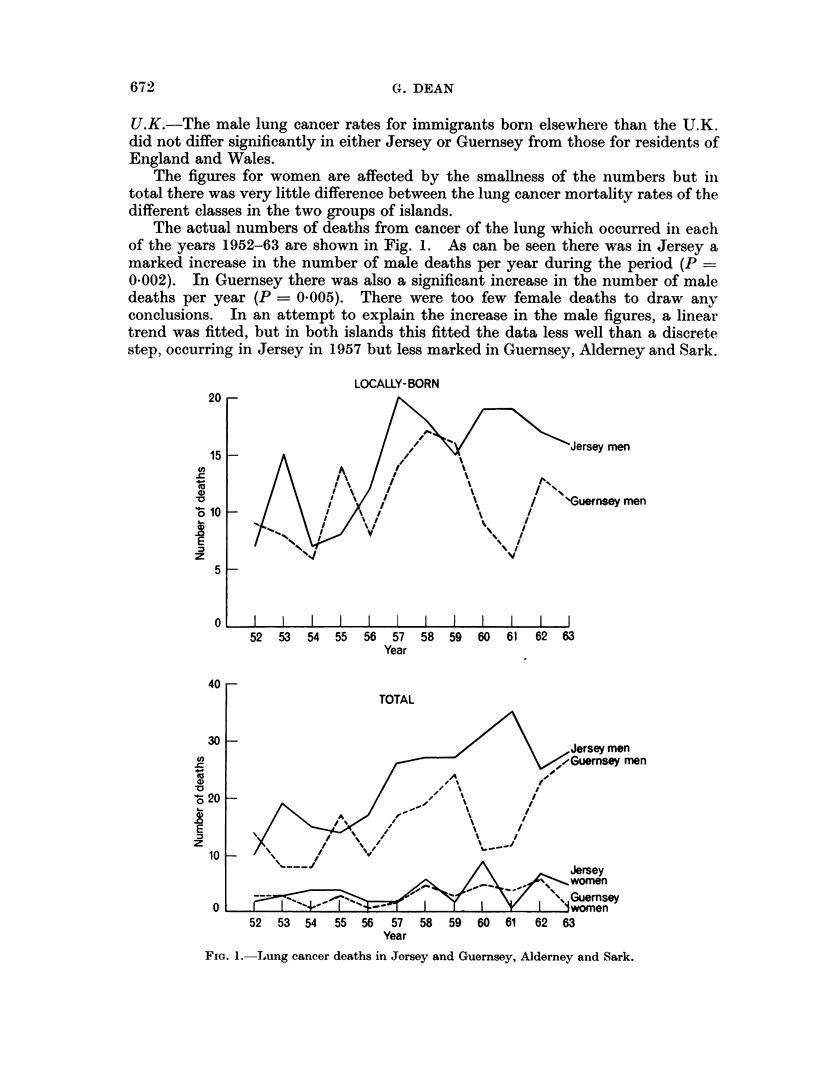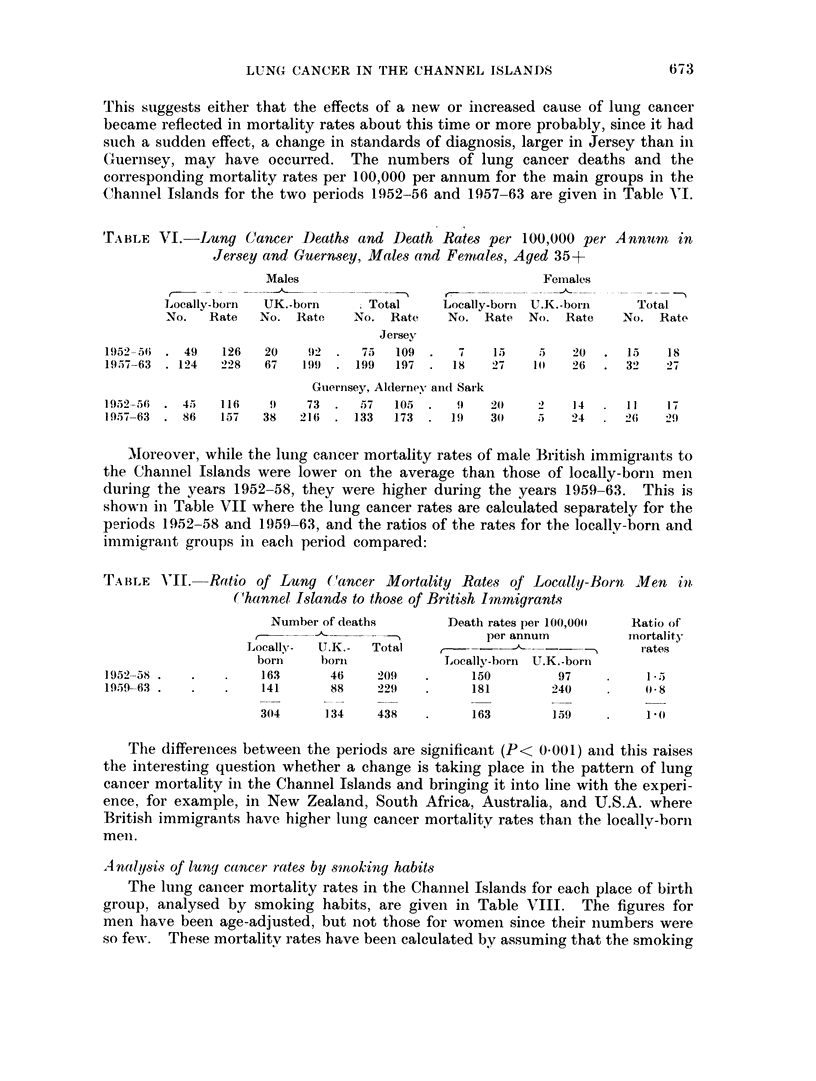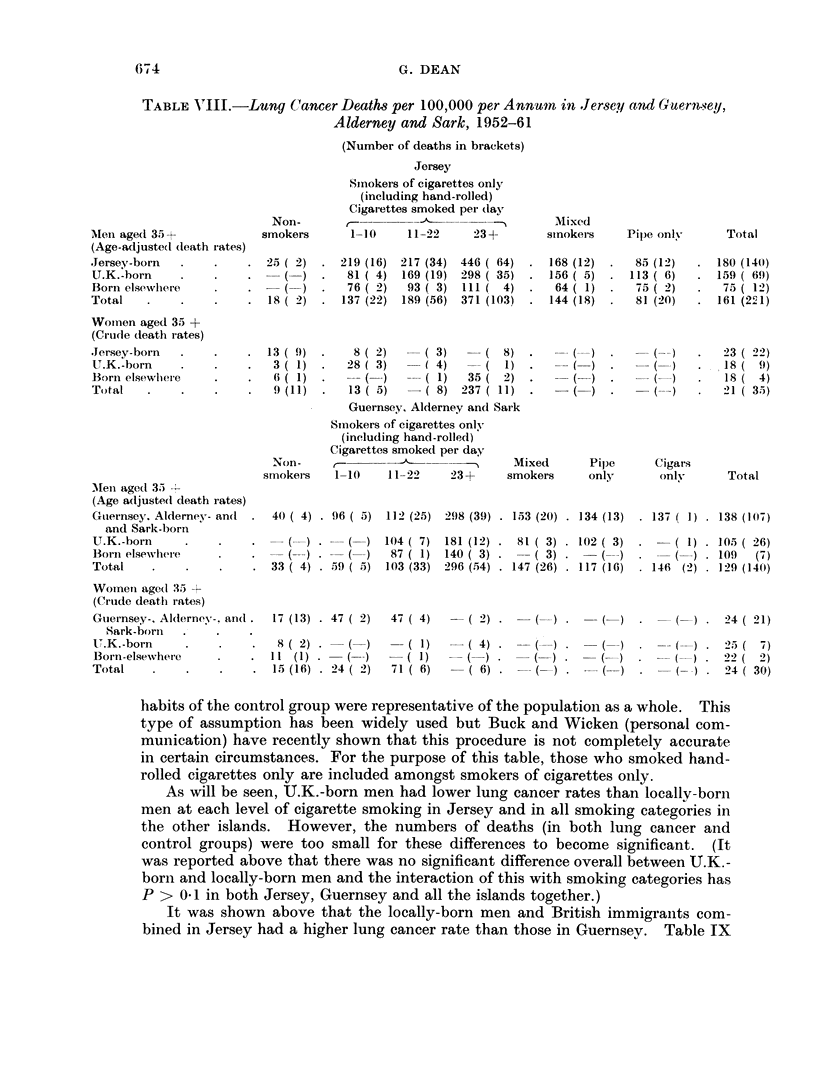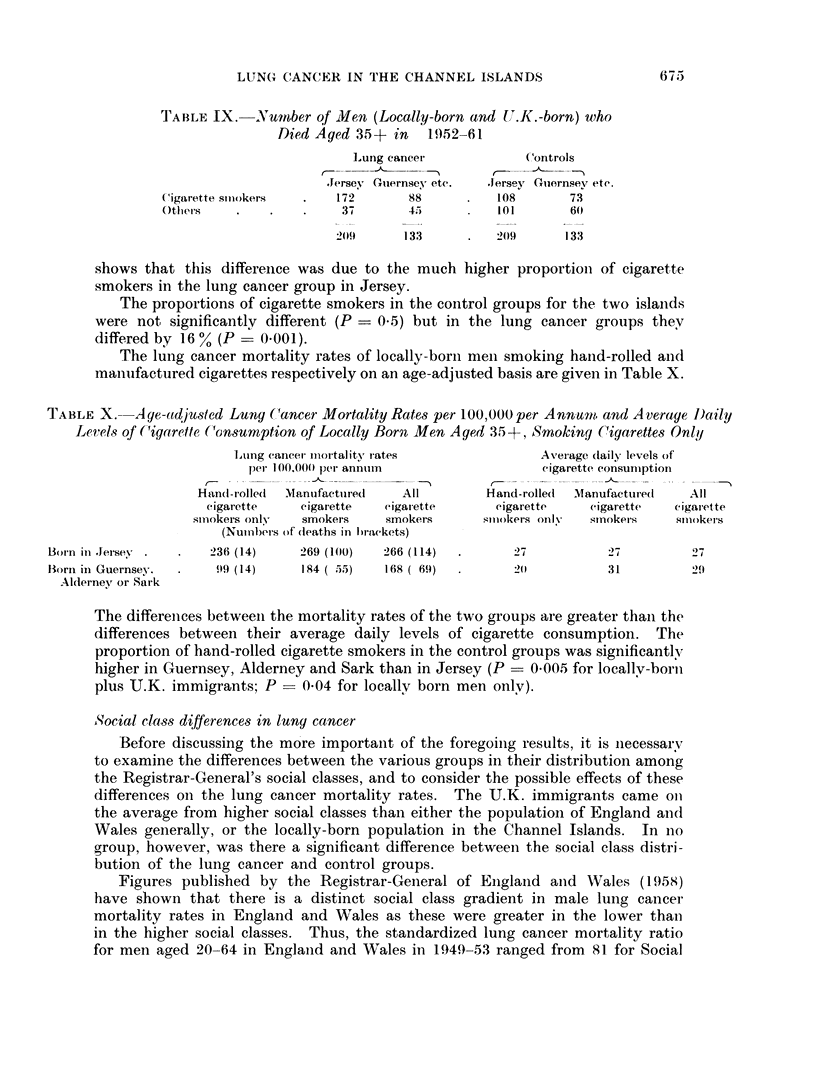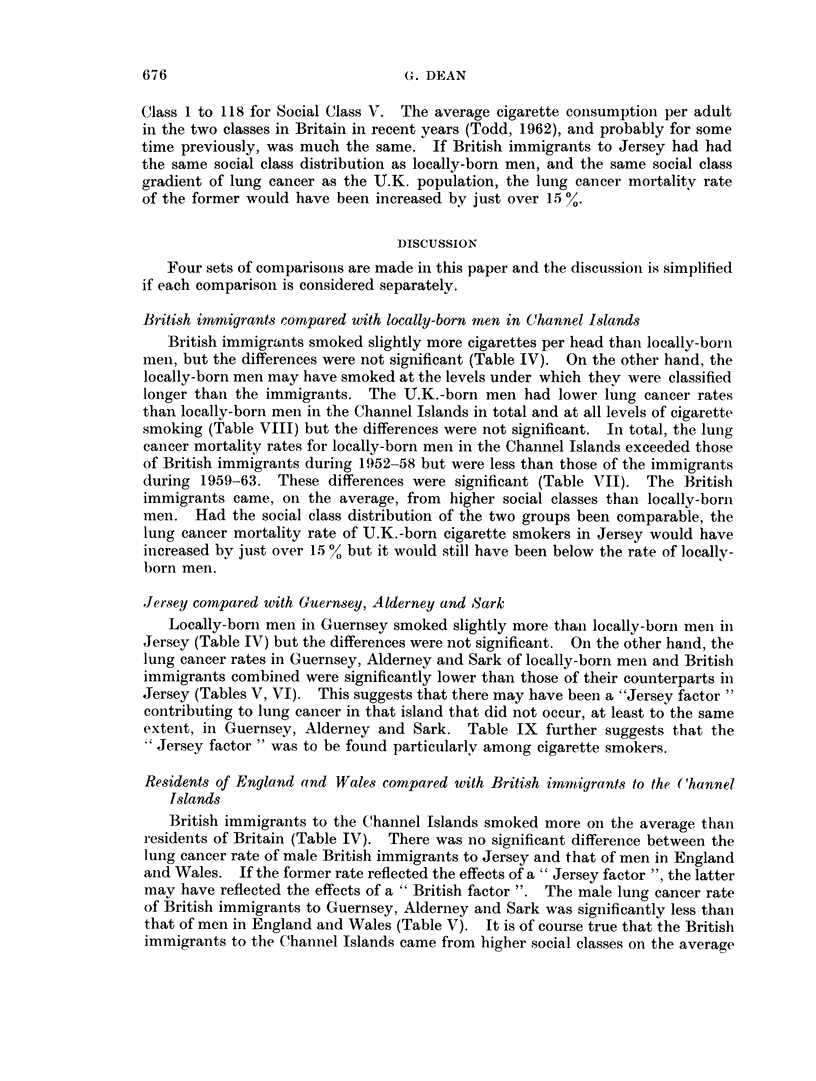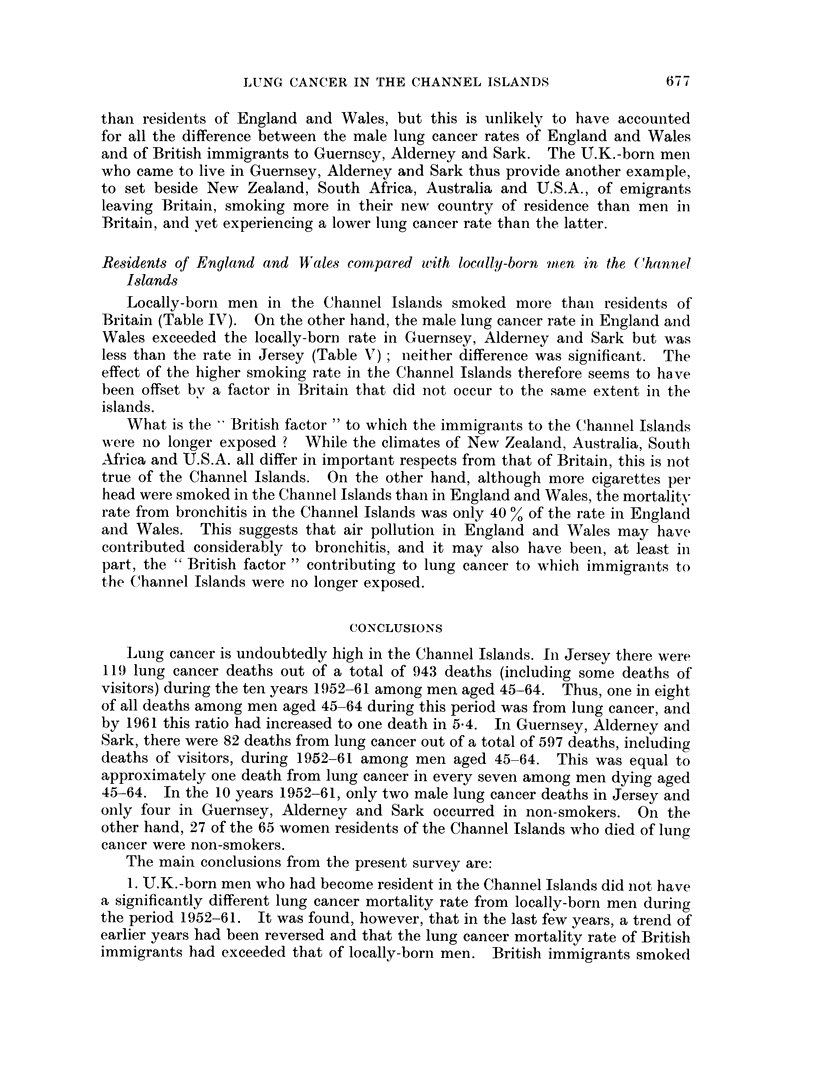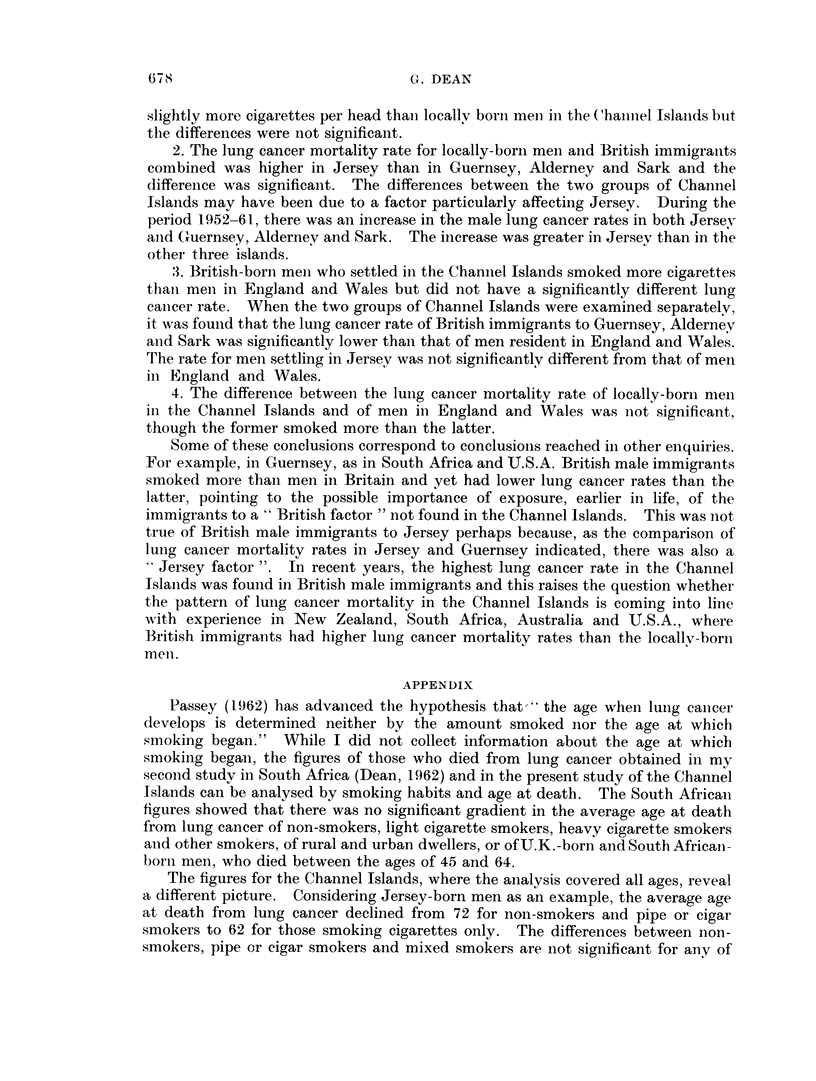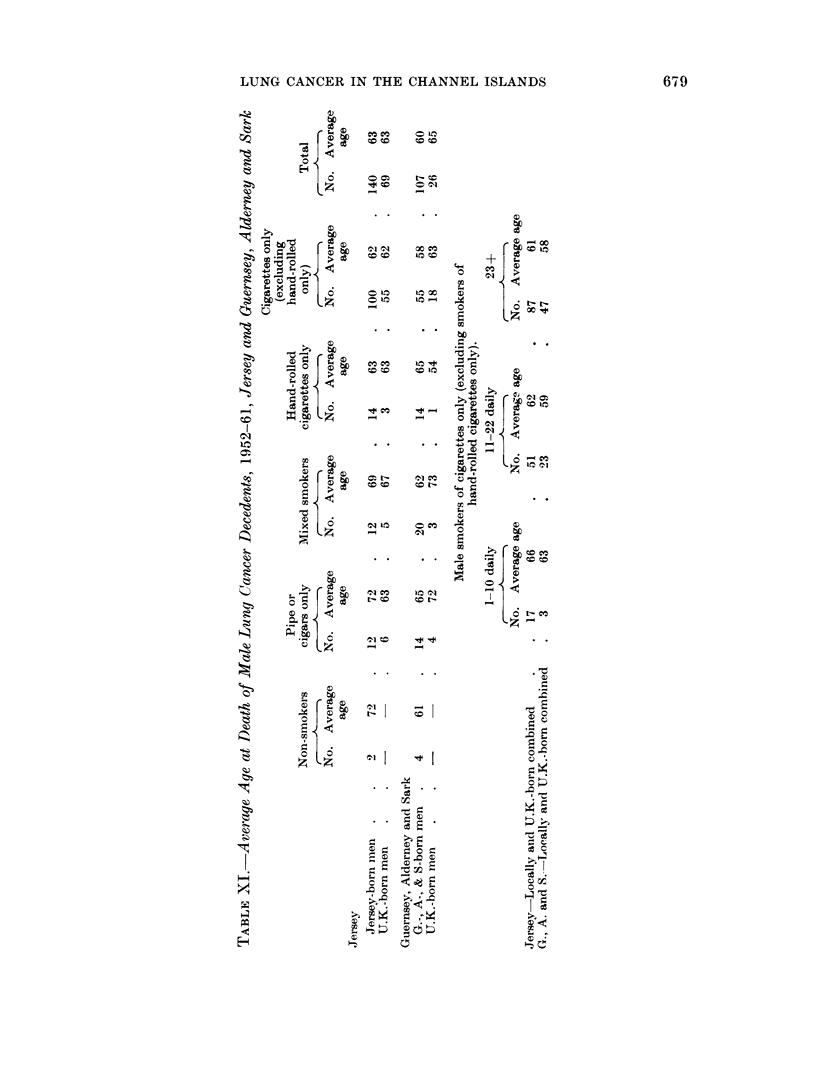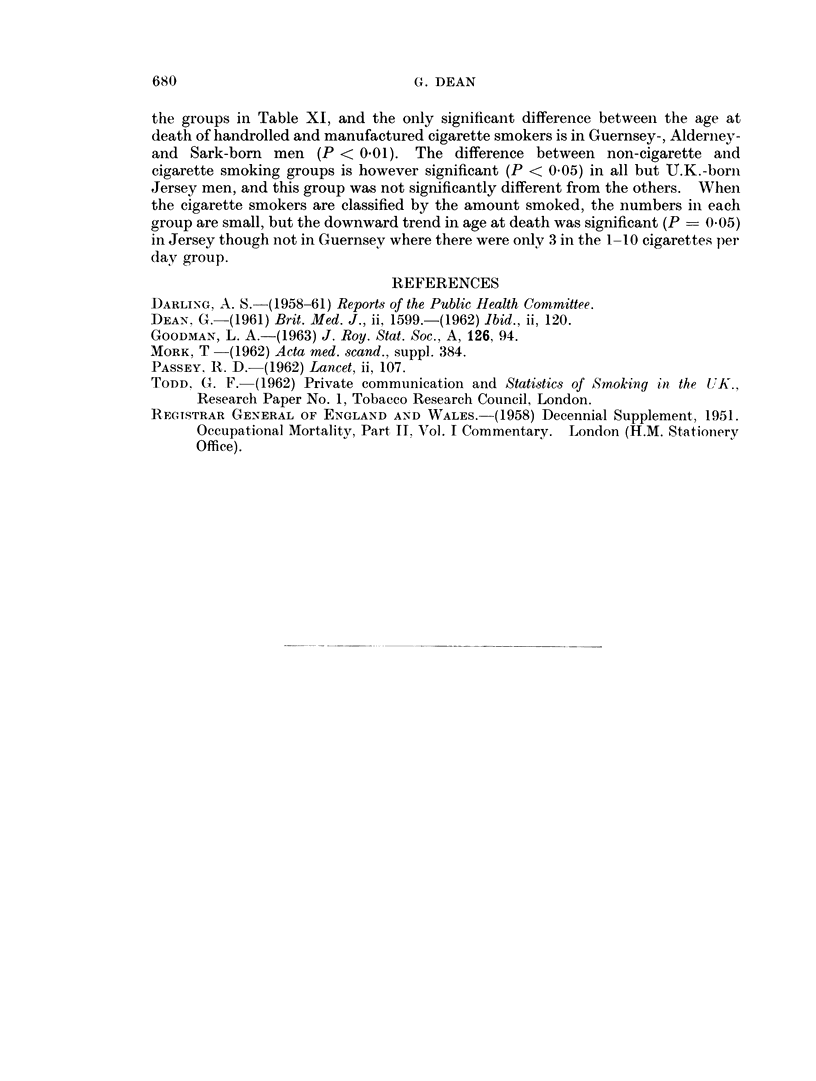# Lung cancer in the Channel Islands.

**DOI:** 10.1038/bjc.1965.81

**Published:** 1965-12

**Authors:** G. Dean


### LUNG CANCER IN THE CHANNEL ISLANDS

G. DEAN

*Eastern Cape Provincial Hospital, Port Elizabeth, South Africa*

Received for publication July 13, 1965

DR A. S. DARLING, while Medical Officer of Health of Jersey, reported (1958–61) that the lung cancer mortality rate in Jersey was high. The population of the Channel Islands in the lung cancer age groups, however, has contained two major groups who may have had substantially different environmental backgrounds—those born in the Channel Islands and who had presumably experienced relatively little exposure to air pollution, and those born in the United Kingdom who had eventually settled in the Channel Islands, many of whom would have experienced considerably greater exposure to air pollution while in the U.K.

I visited the Channel Islands during an extended summer vacation in 1962 and carried out an investigation of the place of birth and smoking habits of all men and women who had died from lung cancer in these Islands during the years 1952–61, and of a control group. Since some unavoidable delays arose in completing this report, the numbers of lung cancer deaths in 1962 and 1963 have been included in some tables. I was extremely fortunate in receiving the full co-operation of the local Medical Officers of Health, Dr. A. S. Darling in Jersey, Dr. A. T. G. Thomas in Guernsey, Dr. D. C. Bell in Alderney and Dr. P. E. S. Court in Sark. These doctors gave me assistance in carrying out this investigation that was quite indispensable. The local Medical Officers of Health, of course, are not responsible in any way for the procedure adopted in the investigation, for the analyses carried out or the conclusions drawn, with which indeed they may not necessarily agree.

The Channel Islands had one particular advantage as an area for investigation of lung cancer. The cigarettes smoked, except during the last war, were broadly similar to those smoked in Britain and Ireland. While differences between cigarettes may affect liability to lung cancer, these differences should be minimized in any comparisons of men and women born in the Channel Islands with those born in the U.K.

As in Britain, the total mortality rate in the 45–64 age group in the Channel Islands is much higher among men than women. In addition, the total mortality rate in the 45–64 age group in 1959–61 was higher in Jersey than in Guernsey for both men ($P = 0{\cdot}005$) and women ($P = 0{\cdot}055$). The total mortality rate for men in England and Wales was lower than in Jersey ($P = 0{\cdot}025$) and higher than in Guernsey ($P = 0{\cdot}025$). Men aged 45–64 in 1959–61 resident in Jersey had a significantly higher death rate from lung cancer than men resident in Guernsey ($P = 0{\cdot}02$). Mortality from cirrhosis of the liver in Jersey was high compared with Guernsey for both men ($P = 0{\cdot}01$) and women ($P = 0{\cdot}02$), and also compared with England and Wales ($P = 0{\cdot}02$ for men, and 0·04 for women). The relevant figures are included in Table I.

TABLE I.—*Number of Deaths and Death Rates per* 100,000 *per Annum of Men and Women Aged* 45–64, *in Channel Islands and England and Wales from Certain Causes*

| | Men | | | | | Women | | | | |
|---|---|---|---|---|---|---|---|---|---|---|
| | Jersey 1959–61 | | Guernsey, Alderney and Sark 1959–61 | | England and Wales 1959–61 | Jersey 1959–61 | | Guernsey, Alderney and Sark 1959–61 | | England and Wales 1959–61 |
| Population (1961) | 7,553 | | 5,597 | | 5,681,000 | 8,226 | | 6,035 | | 6,191,000 |
| Death rates from— | No. of deaths | Death rates | No. of deaths | Death rates | Death rates | No. of deaths | Death rates | No. of deaths | Death rates | Death rates |
| Pulmonary tuberculosis (B1, ISC 001–8) | 6 | 26 | 3 | 18 | 20 | 2 | 8 | 3 | 17 | 5 |
| Lung Cancer (ISC 162–3) | 50 | 221 | 20 | 119 | 174 | 6 | 24 | 4 | 22 | 22 |
| Cancer of sites other than the lung | 38 | 168 | 27 | 161 | 202 | 58 | 235 | 47 | 260 | 244 |
| Arteriosclerotic and degenerative Heart Disease (B26, ISC 420–2) | 95 | 419 | 63 | 375 | 387 | 18 | 73 | 16 | 88 | 108 |
| Pneumonia (B31, ISC 490–3) | 13 | 57 | 5 | 30 | 38 | 8 | 32 | 4 | 22 | 21 |
| Bronchitis (B32, ISC 500–2) | 8 | 35 | 7 | 42 | 105 | 3 | 12 | 1 | 6 | 20 |
| Ulcer of stomach and duodenum (B33, ISC 540–5) | 7 | 31 | 1 | 6 | 18 | 2 | 8 | — | — | 5 |
| Cirrhosis of liver (B37, ISC 581) | 8 | 35 | — | — | 6 | 7 | 28 | — | — | 4 |
| Suicide (BE49, E970–9) | 10 | 44 | 2 | 12 | 24 | 5 | 20 | 2 | 11 | 17 |
| All causes | 343 | 1,514 | 191 | 1,138 | 1,340 | 196 | 794 | 114 | 630 | 729 |

*Procedure in the investigation*

In this investigation of lung cancer mortality in the Channel Islands, the procedure followed was similar to that adopted in my second South African investigation (Dean, 1961). The Registers of Death at the Greffe Offices in the Channel Islands were examined and the names of all those who had died from lung cancer during the 10 year period 1952–61, with their age and place of death, were noted. In Jersey, the place of residence and birth place of decedents are not recorded on the death certificates, although they are recorded in the Bailiwick of Guernsey (which comprises Guernsey, Alderney and Sark). It was therefore necessary in Jersey to find out the place of residence from the hospital records when the patient died in hospital. The place of birth was obtained either from the hospital records or more usually from the relatives of the deceased patient. I also examined all the case records available for those who had died of lung cancer.

Visitors to the Islands who had died of lung cancer while there have been excluded from the tabulations in this paper. Among the island residents, the diagnoses appear to have been adequately verified in nearly all the men, but of the 46 lung cancer deaths among women in Jersey, at least 11 were attributed to lung cancer on what would not seem to have been good evidence and the diagnosis of these cases should be regarded as at least doubtful. Eight of these 11 doubtful cases were non-smokers, whereas only 11 of the remaining 35 cases were non-smokers. These 11 deaths have been excluded from this investigation. Four of these doubtful cases occurred during the years 1959–61 and increased the recorded number of deaths in these years by 33 %.

Nevertheless, the standards of diagnosis in the Islands appear to be high. In Jersey, there is a general hospital and a chest hospital, and there is also a chest consultant in the Island. In Guernsey, there is no consultant chest physician, but a high standard of diagnosis prevails among the group of practitioners in the Island. The majority of the lung cancer patients had been examined by bronchoscope and a number of them had been referred to the mainland for further investigation and treatment. Patients in Alderney and Sark were referred to Guernsey or Britain for investigation.

In order to make as certain as possible that no lung cancer deaths had been overlooked, the records of the local hospitals were examined to obtain the names of all those for whom a diagnosis of lung cancer had been made. This was necessary since a number of patients with lung cancer in the Channel Islands had been referred to Southampton or London hospitals for investigation and treatment. Mr. P. M. Payne, Director of the South Metropolitan Cancer Registry, Sutton, Surrey, kindly supplied detailed records of all patients from the Channel Islands who had been diagnosed in the South Metropolitan area as suffering from lung cancer. This Registry covers most of London and the South of England as far west as Dorset, and therefore included the Southampton Chest Hospital where a number of patients suffering from lung cancer had been referred from the Channel Islands. By this means, 55 patients from Jersey, 12 from Guernsey and one from Sark were notified, of whom five from Jersey, four from Guernsey and one from Sark had died in England although they were residents of the Channel Islands. The doctors in practice in the Channel Islands also reported four other patients resident in the Channel Islands who had died in Britain. It is therefore possible to be reasonably confident that practically all residents in the Channel Islands dying during the 10 years ended 1961 and diagnosed as suffering from lung cancer have been included in this survey.

Table II summarizes the lung cancer deaths of residents in each of the Channel Islands during each of the years 1952–63.

Controls for the lung cancer deaths were obtained by taking the next name after each lung cancer death recorded in the Register of Deaths who was of the same sex, fell into the same age group in the tabulations used in this report, and who had not died of lung cancer. It was also essential that each control should fall into the same place of birth category as the lung cancer decedent whom the control was being used to match. This caused difficulty in Jersey, since the place of birth, not being recorded in the Register of Deaths, could only be ascertained either from the hospital records or after the widow or other relative of the decedent had been successfully interviewed. Since many of those initially selected as controls turned out to have a place of birth category that did not match the lung cancer decedent, very much more work was involved in Jersey in obtaining information about the controls than about the lung cancer deaths. In three cases, due to the complete absence of an exactly matching control, it was necessary to take a control in the year after the year of death of the lung cancer patient in order to find a control in the same age and place of birth category. Nevertheless, as will be seen from the tables in this paper, 100 % response was obtained.

I personally called upon the widow or next-of-kin of all the Jersey lung cancer decedents, when this person was resident in the Island, and obtained information about the place of birth and the last regular smoking habits of the deceased prior to death. For the controls in Jersey and for both lung cancer decedents and controls in the Bailiwick of Guernsey, a shortened procedure was adopted. Dr. Darling in Jersey and Dr. Thomas in Guernsey forwarded a letter and questionnaire to the widow or next-of-kin of the decedent about whom information was required. From these questionnaires there was 65 % response. The non-responders had usually not replied because the letter had not been received by the widow or next-of-kin of the decedent, who had changed their address since the patient's death. There was more than 90 % response from those who had received the questionnaire.

A visit was then made to the last known place of residence of those who did not respond until the next-of-kin was traced. Many sources of information were used to trace the relatives of the lung cancer patients and controls, such as enquiry at their last place of employment, the Pensions Department of the States Office, the household directory and the telephone directory. Fortunately, the islands are small and the Health Visitors and Sanitary Officers of the Health Departments know the islanders very well. By these means, it proved possible to trace the relatives of all male and female lung cancer decedents over the 10 years 1952–61 and of their matched controls in Jersey, Guernsey, Alderney and Sark. The required information was eventually obtained in respect of 852 decedents.

Although two different procedures were used to obtain information about the place of birth and the smoking habits of the control group in Jersey and of both the lung cancer and control groups in the Bailiwick of Guernsey, there is no reason to expect that this difference in the method of obtaining information will have seriously affected the results. Nevertheless, the information about the smoking habits given by the Jersey controls was analysed according to the method of

TABLE II.—*Lung Cancer Deaths of Residents in Channel Islands*, 1952–63

| | Males | | | | | Females | | | | |
|---|---|---|---|---|---|---|---|---|---|---|
| Year | Jersey | Guernsey | Alderney | Sark | Guernsey, Alderney and Sark | Jersey | Guernsey | Alderney | Sark | Guernsey, Alderney and Sark |
| 1952 | 10 | 13 | 1 | — | 14 | 2 | 3 | — | — | 3 |
| 1953 | 19 | 8 | — | — | 8 | 3 | 3 | — | — | 3 |
| 1954 | 15 | 8 | — | — | 8 | 4 | 1 | — | — | 1 |
| 1955 | 14 | 16 | 1 | — | 17 | 4 | 2 | 1 | — | 3 |
| 1956 | 17 | 9 | — | 1 | 10 | 2 | 1 | — | — | 1 |
| 1957 | 26 | 17 | — | — | 17 | 2 | 2 | — | — | 2 |
| 1958 | 27 | 19 | — | — | 19 | 6 | 5 | — | — | 5 |
| 1959 | 27 | 19 | 2 | 3 | 24 | 2 | 3 | — | — | 3 |
| 1960 | 31 | 10 | 1 | — | 11 | 9 | 5 | — | — | 5 |
| 1961 | 35 | 11 | 1 | — | 12 | 1 | 3 | — | 1 | 4 |
| 1962 | 25 | 22 | 1 | — | 23 | 7 | 6 | — | — | 6 |
| 1963 | 28 | 27 | — | — | 27 | 5 | 1 | — | — | 1 |
| TOTAL | 274 | 179 | 7 | 4 | 190 | 47 | 35 | 1 | 1 | 37 |

obtaining the information. The differences between the results obtained by the two methods of collecting the information were not statistically significant.

The smoking habits have been classified so as to give figures for those who smoked cigarettes only, pipes only and mixed smokers. As I have stated in a report on one of my South African surveys, I know no meaningful way, in relation to lung cancer, in which cigarette and pipe tobacco can be added together. I therefore thought it preferable to give separately as far as possible those smoking only one type of tobacco, in order that more clear-cut conclusions could be deduced.

The following points arose in applying the definitions mentioned above:

(a) *Non-smokers* include four men who chewed tobacco only.

(b) The category *Manufactured Cigarettes* comprises those who regularly smoked only cigarettes but excludes those who smoked hand-rolled cigarettes only. It therefore includes those who smoked both manufactured and hand-rolled cigarettes. There are also included in this category a few cigarette smokers who occasionally smoked a pipe or cigar.

(c) The category *Hand-Rolled Cigarettes Only* is self-explanatory. Where the weight of tobacco used for hand-rolling was reported, this was converted to the number of cigarettes at the rate of 35 cigarettes per ounce.

(d) The *Mixed* category includes those who smoked manufactured and/or hand-rolled cigarettes together with pipe tobacco and/or cigars regularly. They are classified according to the number of cigarettes they smoked.

(e) The category of *Pipe Only* consists mainly of those who smoked a pipe only although it does include a few who smoked cigars as well as a pipe.

In the Channel Islands and particularly in the Bailiwick of Guernsey there is considerable consumption of hand-rolled cigarettes and these have therefore been recorded separately. In his discussion of respiratory disease in relation to smoking in Britain and Norway, Mork (1962) pointed out that no estimate had been made of the lung cancer mortality rates associated with the smoking of hand-rolled cigarettes. The figures for the Channel Islands provide an opportunity of making estimates of these.

*Tobacco consumption in the Channel Islands*

Figures for cigarette consumption and for total tobacco consumption in Jersey and in Guernsey can be estimated with considerable accuracy from imports of cigarettes and of manufactured and unmanufactured tobacco. A relatively high proportion, however, of the sales of tobacco products in the Channel Islands is made to visitors, and it is difficult to estimate the quantities that are sold to visitors and consumed by local residents respectively. The tobacco trade in both Jersey and Guernsey, however, very kindly made estimates of the total cigarettes and of all tobacco products consumed by residents in these islands in the light of the best information available to them. The estimates of cigarette consumption per adult in Jersey and Guernsey over a number of years, with comparable figures for the U.K., are given in Table III.

These figures show that consumption of manufactured cigarettes per adult resident has long been considerably higher in the Channel Islands than in the U.K., and much the same in Jersey as in Guernsey.

In Table IV, the smoking habits of U.K.-born men and women and of locally-born men and women who died resident in the islands have been estimated for

TABLE III.—*Manufactured Cigarette Consumption per Head by Local Adult Residents in U.K., Jersey and Guernsey*

| | U.K. | U.K. Average | Jersey from 26th March | Jersey Average | Jersey as % of U.K. | Guernsey* | Guernsey Average | Guernsey as % of U.K. |
|---|---|---|---|---|---|---|---|---|
| 1933 | 1,470 | 1,490 | 1,580 | 1,600 | 107 | — | | — |
| 1934 | 1,500 | | 1,610 | | | — | | — |
| 1935 | 1,590 | 1,780 | 1,900 | 2,010 | 113 | 1,800 | 1,950 | 110 |
| 1936 | 1,690 | | 1,760 | | | 1,920 | | |
| 1937 | 1,790 | | 2,050 | | | 1,960 | | |
| 1938 | 1,880 | | 2,070 | | | 2,110 | | |
| 1939 | 1,970 | | 2,250 | | | — | | |
| 1946 | 2,600 | 2,290 | 3,000 | 3,670 | 160 | 3,130 | 3,310 | 145 |
| 1947 | 2,270 | | 3,870 | | | 3,380 | | |
| 1948 | 2,170 | | 4,470 | | | 3,750 | | |
| 1949 | 2,110 | | 3,320 | | | 2,960 | | |
| 1950 | 2,180 | 2,320 | 2,920 | 3,040 | 131 | 2,730 | 2,950 | 127 |
| 1951 | 2,300 | | 3,280 | | | 3,110 | | |
| 1952 | 2,320 | | 2,870 | | | 2,950 | | |
| 1953 | 2,370 | | 3,160 | | | 2,900 | | |
| 1954 | 2,430 | | 2,980 | | | 3,060 | | |
| 1955 | 2,510 | 2,590 | 2,950 | 3,020 | 117 | 3,380 | 3,220 | 124 |
| 1956 | 2,530 | | 3,050 | | | 2,210 | | |
| 1957 | 2,590 | | 2,940 | | | 3,210 | | |
| 1958 | 2,630 | | 3,030 | | | 3,170 | | |
| 1959 | 2,670 | | 3,140 | | | 3,110 | | |
| 1960 | 2,760 | 2,790 | 3,230 | 3,150 | 113 | 3,150 | 3,190 | 114 |
| 1961 | 2,810 | | 3,060 | | | 3,220 | | |

* The figures for Guernsey exclude Alderney and Sark.

three age groups for men and in total for women from figures obtained from my control groups and compared with those of residents of Britain given by Todd (1962).

The percentage of men resident in the U.K. who smoked cigarettes only and at the rate of 11 or more cigarettes a day were less than the corresponding percentages among the U.K.-born men in both Jersey and Guernsey in all three age groups (i.e. 45–59, 60–69 and 70+) and less than the corresponding percentages among the locally-born in the Channel Islands in the 45–59 and 60–69 age groups, although the differences in the 45–59 age group were relatively small. There was no similar pattern in women but the numbers involved were too few to enable reliable conclusions to be drawn.

In his report for 1958 and also for subsequent years, the Medical Officer of Health in Jersey published estimates of the distribution of smoking habits in Jersey based upon questionnaires completed by many of those attending the Mass Miniature Radiography Centre. I reclassified the figures obtained from my Jersey control group so as to provide as direct a comparison as possible with the 1958 figures of Dr. Darling. The results showed that, although there was a higher percentage of pipe smokers in my controls in Jersey than in Dr. Darling's figures, there was otherwise no significant difference between Dr. Darling's figures and mine for the distribution of male cigarette smokers among the different levels of smoking. The difference in pipe smokers was mainly due to a much larger proportion of older men in my figures. For the purpose of this reclassification, mixed smokers among my controls were divided between pipe and cigarette smoking

TABLE IV.—*Distribution of Smoking Habits (U.K. 1956 : Channel Islands 1952–61)*

Men

Aged 45–59

| | U.K. 1956 % | U.K.-born Jersey No. | U.K.-born Jersey % | Jersey-born No. | Jersey-born % | U.K.-born Guernsey No. | U.K.-born Guernsey % | Guernsey-born No. | Guernsey-born % |
|---|---|---|---|---|---|---|---|---|---|
| Non-smokers | 20 | 1 | 4 | 9 | 18 | — | — | 7 | 15 |
| Cigarettes only— | | | | | | | | | |
| 1–10 per day | 18 | 3 | 12 | 7 | 14 | 2 | 22 | 5 | 11 |
| 11–22 per day | 25 | 9 | 36 | 13 | 27 | 4 | 45 | 15 | 32 |
| 23 + per day | 18 | 10 | 40 | 10 | 21 | 1 | 11 | 10 | 21 |
| Pipe only or cigars only | 9 | 1 | 4 | 5 | 10 | — | — | 2 | 4 |
| Mixed smokers | 10 | 1 | 4 | 5 | 10 | 2 | 22 | 8 | 17 |
| Total | 100 | 25 | 100 | 49 | 100 | 9 | 100 | 47 | 100 |

Aged 60–69

| | U.K. 1956 % | U.K.-born Jersey No. | U.K.-born Jersey % | Jersey-born No. | Jersey-born % | U.K.-born Guernsey No. | U.K.-born Guernsey % | Guernsey-born No. | Guernsey-born % |
|---|---|---|---|---|---|---|---|---|---|
| Non-smokers | 21 | 4 | 21 | 9 | 19 | 1 | 12 | 4 | 10 |
| Cigarettes only— | | | | | | | | | |
| 1–10 per day | 20 | 3 | 16 | 4 | 8 | — | — | 2 | 5 |
| 11–22 per day | 19 | 6 | 31 | 12 | 25 | 2 | 22 | 15 | 37 |
| 23 + per day | 9 | 3 | 16 | 12 | 25 | 2 | 22 | 6 | 15 |
| Pipe only or cigars only | 21 | 3 | 16 | 5 | 10 | 2 | 22 | 6 | 15 |
| Mixed smokers | 10 | — | — | 6 | 13 | 2 | 22 | 7 | 18 |
| Total | 100 | 19 | 100 | 48 | 100 | 9 | 100 | 40 | 100 |

Aged 70 +

| | U.K. 1956 % | U.K.-born Jersey No. | U.K.-born Jersey % | Jersey-born No. | Jersey-born % | U.K.-born Guernsey No. | U.K.-born Guernsey % | Guernsey-born No. | Guernsey-born % |
|---|---|---|---|---|---|---|---|---|---|
| Non-smokers | 22 | 3 | 13 | 12 | 31 | 1 | 13 | 5 | 29 |
| Cigarettes only— | | | | | | | | | |
| 1–10 per day | 12 | 4 | 17 | 3 | 8 | 2 | 25 | 2 | 12 |
| 11–22 per day | 11 | 3 | 12 | 1 | 3 | 1 | 12 | — | — |
| 23 + per day | 5 | 2 | 8 | 1 | 2 | 2 | 25 | 1 | 6 |
| Pipe only or cigars only | 38 | 8 | 33 | 18 | 46 | 1 | 13 | 6 | 35 |
| Mixed smokers | 12 | 4 | 17 | 4 | 10 | 1 | 12 | 3 | 18 |
| Total | 100 | 24 | 100 | 39 | 100 | 8 | 100 | 17 | 100 |

Total aged 35 +

| | U.K. 1956 % | U.K.-born Jersey No. | U.K.-born Jersey % | Jersey-born No. | Jersey-born % | U.K.-born Guernsey No. | U.K.-born Guernsey % | Guernsey-born No. | Guernsey-born % |
|---|---|---|---|---|---|---|---|---|---|
| Non-smokers | 21 | 8 | 12 | 33 | 24 | 2 | 8 | 18 | 17 |
| Cigarettes only— | | | | | | | | | |
| 1–10 per day | 19 | 10 | 15 | 14 | 10 | 4 | 15 | 9 | 8 |
| 11–22 per day | 19 | 18 | 26 | 26 | 19 | 7 | 27 | 31 | 29 |
| 23 + per day | 9 | 16 | 23 | 24 | 16 | 5 | 19 | 17 | 16 |
| Pipe only or cigars only | 17 | 12 | 17 | 28 | 20 | 3 | 12 | 14 | 13 |
| Mixed smokers | 15 | 5 | 7 | 15 | 11 | 5 | 19 | 18 | 17 |
| Total | 100 | 69 | 100 | 140 | 100 | 26 | 100 | 107 | 100 |

Women

Total aged 35 +

| | U.K. 1956 % | U.K.-born Jersey No. | U.K.-born Jersey % | Jersey-born No. | Jersey-born % | U.K.-born Guernsey No. | U.K.-born Guernsey % | Guernsey-born No. | Guernsey-born % |
|---|---|---|---|---|---|---|---|---|---|
| Non-smokers | 61 | 7 | 78 | 16 | 73 | 6 | 86 | 18 | 86 |
| Cigarettes only— | | | | | | | | | |
| 1–10 per day | 28 | 2 | 22 | 6 | 27 | 1 | 14 | 1 | 5 |
| 11–22 per day | 9 | — | — | — | — | — | — | 2 | 9 |
| 23 + per day | 2 | — | — | — | — | — | — | — | — |
| Total | 100 | 9 | 100 | 22 | 100 | 7 | 100 | 21 | 100 |

according to the form in which they mainly smoked tobacco. Dr. Darling had very few mixed smokers among those who completed questionnaires and those were classified according to the form in which they smoked most tobacco and on the basis of the total tobacco they smoked. There were also significant differences between the two groups of women due to a higher proportion of non-smokers in my controls. A greater proportion in my 55+ age group explains this difference.

It is not unexpected that, as the figures in Table IV suggest, British immigrants to the Channel Islands should have smoked more than men in Britain. Cigarettes are relatively cheap in the Channel Islands, comparable brands selling until about mid-1962 at 20 cigarettes for 1/8½d. in Jersey, 1/9d. in Guernsey and Alderney, 1/7d. in Sark and 4/6d. in Great Britain. There was therefore a strong inducement to the immigrants to increase their tobacco consumption. On the other hand, in calculating the lung cancer mortality rates at the various levels of smoking shown later in Table VIII, people were classified according to their last regular smoking habits prior to their death. It is possible, therefore, that those born in the islands were classified according to a level of smoking that they may have smoked over a considerably longer period than the British immigrants classified under the same levels of smoking.

There is another factor peculiar to smoking in the Channel Islands. During the war, no tobacco was imported into the Channel Islands and smokers were forced to smoke substitutes from the gardens, fields or woods. The most popular substitutes, in approximate order of magnitude, were cherry leaves, sweet chestnut leaves, rose leaves, sweet scented butterberry, coltsfoot and clover. It is not inconceivable that these substitutes may have made some contribution to the eventual development of lung cancer.

Analyses of the population of the Channel Islands by island of residence, place of birth and age have not previously been published. The Registrar-General of England and Wales, however, has supplied the Medical Officers of Health in the islands with analyses of the 1961 Census figures on this basis. The lung cancer mortality figures covered the 10 years 1952–61, and accordingly the population figures required for estimation of the lung cancer mortality rates are the averages for this period. A comparison of the population of the Channel Islands according to the 1951 and 1961 Censuses showed that the main changes between the Census years were increases of 25 % and 24 % respectively in the male and female residents of Jersey who had been born in the U.K., and an increase of 33 % in male residents of Jersey who had been born elsewhere than Jersey or the U.K. Although details of the population of the Channel Islands within each age group were available from the 1961 Census, there were no comparable figures from the 1951 Census except in total by island of residence and place of birth. In calculating the lung cancer mortality rates, therefore, it was assumed that the change that occurred in the total figures for each sex applied also to each age group of the sex, and when calculating mortality rates for the period 1952–61 the mean population between the two census years was used.

### RESULTS

### *Lung cancer mortality rates*

Throughout this paper, where comparisons of lung cancer deaths have been made, an approach put forward by Goodman (1963, page 99) has been used. This is based on the ratio of the lung cancer mortality in one area to that in another. The ratios for individual five-year age groups were suitably averaged to eliminate age differences and a test given by Goodman was used to assess the significance of the departure of the mean mortality ratio from unity. Table V summarizes the lung cancer mortality rates per 100,000 per annum for locally-born, U.K.-born and others in Jersey, in Guernsey, Alderney and Sark and in all the islands combined for each age-group for the period 1952–61. Corresponding figures for England and Wales for 1952–61 are included in Table V.

As far as men are concerned, the figures in Table V lead to the following conclusions:

*British immigrants compared with locally-born men in Channel Islands.*—The main hypothesis which it is desired to test is whether U.K.-born men who had become resident in the Channel Islands had a higher lung cancer mortality rate than island-born men. Taking the Channel Islands as a whole, the lung cancer mortality rates of the two groups of men were not significantly different. It is natural to ask, however, whether the same conclusion is also true of the two groups of islands. U.K.-born men had lower lung cancer mortality rates than locally-born men in Jersey and in Guernsey, Alderney and Sark, but the mortality ratios of the two groups were not significantly different from 1.

*Jersey compared with Guernsey, Alderney and Sark.*—Although the lung cancer mortality rates of the U.K.-born immigrants were not significantly different from the rates of locally-born men it was of interest to investigate whether any differences existed in the mortality rates for men between the two groups of islands. There were no significant differences between Jersey and the other islands if the locally-born group or the immigrant groups are considered separately (locally-born $P = 0{\cdot}07$, U.K.-born $P = 0{\cdot}09$). If, however, the locally-born and U.K.-born residents are combined, the rate for Jersey was significantly higher than the rate for the other islands ($P = 0{\cdot}01$).

*Residents of England and Wales compared with British immigrants to the Channel Islands.*—The next question of interest is whether men born in the U.K. who eventually became residents of the Channel Islands had a significantly different lung cancer mortality rate from men resident in England and Wales. The answer is that the difference between the rates of the two groups was not significant. Next, considering the two groups of Channel Islands separately, in all but two age groups in Jersey, the lung cancer mortality rate was greater for residents of England and Wales than for U.K.-born immigrants to Jersey. The difference, however, did not reach the 5 % significance level. In Guernsey, the rate for U.K.-born immigrants was lower in every age group than the rate for residents of England and Wales. This difference was significant ($P = 0{\cdot}04$).

*Residents of England and Wales compared with locally-born men in the Channel Islands.*—The locally-born men in Jersey had a higher lung cancer rate than men in England and Wales. In Guernsey, locally born men had a lower rate than men in England and Wales. Neither difference approached the 5 % significance level.

*Locally-born men in Channel Islands compared with immigrants born elsewhere than U.K.*—Residents of the Channel Islands born outside the islands elsewhere than the U.K. had lower lung cancer rates than the locally-born ($P = 0{\cdot}03$). The differences between corresponding figures for Jersey and Guernsey separately were not significant.

*Residents of England and Wales compared with immigrants born elsewhere than*

TABLE V.—*Lung Cancer Mortality Rates per* 100,000 *per Annum by Age Groups* 1952–61

(Numbers of deaths in brackets)

| Age | All Channel Islands | | | Jersey | | | Guernsey, Alderney and Sark | | | England and Wales |
|---|---|---|---|---|---|---|---|---|---|---|
| | Locally-born | U.K.-born | Born elsewhere | Locally-born | U.K.-born | Born elsewhere | Locally-born | U.K.-born | Born elsewhere | |
| Men | | | | | | | | | | |
| 35–44 | 17 ( 7) | 6 ( 1) | — (—) | 20 ( 4) | 10 ( 1) | — (—) | 15 ( 3) | — (—) | — (—) | 17 |
| 45–49 | 74 ( 16) | 92 ( 9) | 90 ( 3) | 65 ( 7) | 123 ( 8) | 127 ( 3) | 84 ( 9) | 30 ( 1) | — (—) | 58 |
| 50–54 | 142 ( 31) | 75 ( 8) | 64 ( 2) | 133 ( 14) | 83 ( 6) | 84 ( 2) | 150 ( 17) | 58 ( 2) | — (—) | 124 |
| 55–59 | 249 ( 49) | 198 (17) | 110 ( 3) | 287 ( 28) | 201 (11) | 98 ( 2) | 212 ( 21) | 192 ( 6) | 147 ( 1) | 228 |
| 60–64 | 262 ( 43) | 213 (16) | 216 ( 4) | 286 ( 25) | 261 (12) | 75 ( 1) | 234 ( 18) | 137 ( 4) | 588 ( 3) | 317 |
| 65–69 | 355 ( 45) | 211 (12) | 174 ( 3) | 357 ( 23) | 188 ( 7) | — (—) | 353 ( 22) | 256 ( 5) | 484 ( 3) | 366 |
| 70 + | 251 ( 56) | 371 (32) | 130 ( 4) | 348 ( 39) | 458 (24) | 227 ( 4) | 154 ( 17) | 236 ( 8) | — (—) | 298 |
| Total | 159 (247) | 140 (95) | 85 (19) | 180 (140) | 159 (69) | 75 (12) | 138 (107) | 105 (26) | 109 ( 7) | 151 |
| Women | | | | | | | | | | |
| 35–44 | 7 ( 3) | 5 ( 1) | — (—) | 5 ( 1) | 8 ( 1) | — (—) | 10 ( 2) | — (—) | — (—) | 4 |
| 45–49 | 9 ( 2) | — (—) | 26 ( 1) | 17 ( 2) | — (—) | 34 ( 1) | — (—) | — (—) | — (—) | 10 |
| 50–54 | 23 ( 5) | — (—) | 52 ( 2) | 27 ( 3) | — (—) | 34 ( 1) | 19 ( 2) | — (—) | 105 ( 1) | 16 |
| 55–59 | 42 ( 9) | 54 ( 5) | — (—) | 38 ( 4) | 52 ( 3) | — (—) | 45 ( 5) | 58 ( 2) | — (—) | 24 |
| 60–64 | 25 ( 5) | 72 ( 6) | — (—) | 29 ( 3) | 38 ( 2) | — (—) | 21 ( 2) | 129 ( 4) | — (—) | 32 |
| 65–69 | 17 ( 3) | 48 ( 3) | 32 ( 1) | 22 ( 2) | 52 ( 2) | 47 ( 1) | 12 ( 1) | 42 ( 1) | — (—) | 38 |
| 70 + | 42 (16) | 8 ( 1) | 25 ( 2) | 34 ( 7) | 13 ( 1) | 20 ( 1) | 50 ( 9) | — (—) | 34 ( 1) | 45 |
| Total | 23 (43) | 21 (16) | 19 ( 6) | 23 (22) | 18 ( 9) | 18 ( 4) | 24 (21) | 25 ( 7) | 22 ( 2) | 21 |

*U.K.*—The male lung cancer rates for immigrants born elsewhere than the U.K. did not differ significantly in either Jersey or Guernsey from those for residents of England and Wales.

The figures for women are affected by the smallness of the numbers but in total there was very little difference between the lung cancer mortality rates of the different classes in the two groups of islands.

The actual numbers of deaths from cancer of the lung which occurred in each of the years 1952–63 are shown in Fig. 1. As can be seen there was in Jersey a marked increase in the number of male deaths per year during the period ($P = 0{\cdot}002$). In Guernsey there was also a significant increase in the number of male deaths per year ($P = 0{\cdot}005$). There were too few female deaths to draw any conclusions. In an attempt to explain the increase in the male figures, a linear trend was fitted, but in both islands this fitted the data less well than a discrete step, occurring in Jersey in 1957 but less marked in Guernsey, Alderney and Sark.

FIG. 1.—Lung cancer deaths in Jersey and Guernsey, Alderney and Sark.

This suggests either that the effects of a new or increased cause of lung cancer became reflected in mortality rates about this time or more probably, since it had such a sudden effect, a change in standards of diagnosis, larger in Jersey than in Guernsey, may have occurred. The numbers of lung cancer deaths and the corresponding mortality rates per 100,000 per annum for the main groups in the Channel Islands for the two periods 1952–56 and 1957–63 are given in Table VI.

TABLE VI.—*Lung Cancer Deaths and Death Rates per* 100,000 *per Annum in Jersey and Guernsey, Males and Females, Aged* 35+

| | Males | | | | | | Females | | | | | |
|---|---|---|---|---|---|---|---|---|---|---|---|---|
| | Locally-born | | UK.-born | | Total | | Locally-born | | U.K.-born | | Total | |
| | No. | Rate | No. | Rate | No. | Rate | No. | Rate | No. | Rate | No. | Rate |
| Jersey | | | | | | | | | | | | |
| 1952–56 | 49 | 126 | 20 | 92 | 75 | 109 | 7 | 15 | 5 | 20 | 15 | 18 |
| 1957–63 | 124 | 228 | 67 | 199 | 199 | 197 | 18 | 27 | 10 | 26 | 32 | 27 |
| Guernsey, Alderney and Sark | | | | | | | | | | | | |
| 1952–56 | 45 | 116 | 9 | 73 | 57 | 105 | 9 | 20 | 2 | 14 | 11 | 17 |
| 1957–63 | 86 | 157 | 38 | 216 | 133 | 173 | 19 | 30 | 5 | 24 | 26 | 29 |

Moreover, while the lung cancer mortality rates of male British immigrants to the Channel Islands were lower on the average than those of locally-born men during the years 1952–58, they were higher during the years 1959–63. This is shown in Table VII where the lung cancer rates are calculated separately for the periods 1952–58 and 1959–63, and the ratios of the rates for the locally-born and immigrant groups in each period compared:

TABLE VII.—*Ratio of Lung Cancer Mortality Rates of Locally-Born Men in Channel Islands to those of British Immigrants*

| | Number of deaths | | | Death rates per 100,000 per annum | | Ratio of mortality rates |
|---|---|---|---|---|---|---|
| | Locally-born | U.K.-born | Total | Locally-born | U.K.-born | |
| 1952–58 | 163 | 46 | 209 | 150 | 97 | 1·5 |
| 1959–63 | 141 | 88 | 229 | 181 | 240 | 0·8 |
| | 304 | 134 | 438 | 163 | 159 | 1·0 |

The differences between the periods are significant ($P < 0{\cdot}001$) and this raises the interesting question whether a change is taking place in the pattern of lung cancer mortality in the Channel Islands and bringing it into line with the experience, for example, in New Zealand, South Africa, Australia, and U.S.A. where British immigrants have higher lung cancer mortality rates than the locally-born men.

*Analysis of lung cancer rates by smoking habits*

The lung cancer mortality rates in the Channel Islands for each place of birth group, analysed by smoking habits, are given in Table VIII. The figures for men have been age-adjusted, but not those for women since their numbers were so few. These mortality rates have been calculated by assuming that the smoking

TABLE VIII.—*Lung Cancer Deaths per* 100,000 *per Annum in Jersey and Guernsey, Alderney and Sark*, 1952–61

(Number of deaths in brackets)

Jersey

| | Non-smokers | Smokers of cigarettes only (including hand-rolled) Cigarettes smoked per day 1–10 | 11–22 | 23+ | Mixed smokers | Pipe only | Total |
|---|---|---|---|---|---|---|---|
| Men aged 35+ (Age-adjusted death rates) | | | | | | | |
| Jersey-born | 25 ( 2) | 219 (16) | 217 (34) | 446 ( 64) | 168 (12) | 85 (12) | 180 (140) |
| U.K.-born | — (—) | 81 ( 4) | 169 (19) | 298 ( 35) | 156 ( 5) | 113 ( 6) | 159 ( 69) |
| Born elsewhere | — (—) | 76 ( 2) | 93 ( 3) | 111 ( 4) | 64 ( 1) | 75 ( 2) | 75 ( 12) |
| Total | 18 ( 2) | 137 (22) | 189 (56) | 371 (103) | 144 (18) | 81 (20) | 161 (221) |
| Women aged 35 + (Crude death rates) | | | | | | | |
| Jersey-born | 13 ( 9) | 8 ( 2) | — ( 3) | — ( 8) | — (—) | — (—) | 23 ( 22) |
| U.K.-born | 3 ( 1) | 28 ( 3) | — ( 4) | — ( 1) | — (—) | — (—) | 18 ( 9) |
| Born elsewhere | 6 ( 1) | — (—) | — ( 1) | 35 ( 2) | — (—) | — (—) | 18 ( 4) |
| Total | 9 (11) | 13 ( 5) | — ( 8) | 237 ( 11) | — (—) | — (—) | 21 ( 35) |

Guernsey, Alderney and Sark

| | Non-smokers | Smokers of cigarettes only (including hand-rolled) Cigarettes smoked per day 1–10 | 11–22 | 23+ | Mixed smokers | Pipe only | Cigars only | Total |
|---|---|---|---|---|---|---|---|---|
| Men aged 35 + (Age adjusted death rates) | | | | | | | | |
| Guernsey, Alderney- and and Sark-born | 40 ( 4) | 96 ( 5) | 112 (25) | 298 (39) | 153 (20) | 134 (13) | 137 ( 1) | 138 (107) |
| U.K.-born | — (—) | — (—) | 104 ( 7) | 181 (12) | 81 ( 3) | 102 ( 3) | — ( 1) | 105 ( 26) |
| Born elsewhere | — (—) | — (—) | 87 ( 1) | 140 ( 3) | — ( 3) | — (—) | — (—) | 109 (7) |
| Total | 33 ( 4) | 59 ( 5) | 103 (33) | 296 (54) | 147 (26) | 117 (16) | 146 (2) | 129 (140) |
| Women aged 35 + (Crude death rates) | | | | | | | | |
| Guernsey-, Alderney-, and Sark-born | 17 (13) | 47 ( 2) | 47 ( 4) | — ( 2) | — (—) | — (—) | — (—) | 24 ( 21) |
| U.K.-born | 8 ( 2) | — (—) | — ( 1) | — ( 4) | — (—) | — (—) | — (—) | 25 ( 7) |
| Born-elsewhere | 11 (1) | — (—) | — ( 1) | — (—) | — (—) | — (—) | — (—) | 22 ( 2) |
| Total | 15 (16) | 24 ( 2) | 71 ( 6) | — ( 6) | — (—) | — (—) | — (—) | 24 ( 30) |

habits of the control group were representative of the population as a whole. This type of assumption has been widely used but Buck and Wicken (personal communication) have recently shown that this procedure is not completely accurate in certain circumstances. For the purpose of this table, those who smoked hand-rolled cigarettes only are included amongst smokers of cigarettes only.

As will be seen, U.K.-born men had lower lung cancer rates than locally-born men at each level of cigarette smoking in Jersey and in all smoking categories in the other islands. However, the numbers of deaths (in both lung cancer and control groups) were too small for these differences to become significant. (It was reported above that there was no significant difference overall between U.K.-born and locally-born men and the interaction of this with smoking categories has $P > 0{\cdot}1$ in both Jersey, Guernsey and all the islands together.)

It was shown above that the locally-born men and British immigrants combined in Jersey had a higher lung cancer rate than those in Guernsey. Table IX

TABLE IX.—*Number of Men (Locally-born and U.K.-born) who Died Aged* 35+ *in* 1952–61

| | Lung cancer | | | Controls | |
|---|---|---|---|---|---|
| | Jersey | Guernsey etc. | | Jersey | Guernsey etc. |
| Cigarette smokers . | 172 | 88 | . | 108 | 73 |
| Others . . . | 37 | 45 | . | 101 | 60 |
| | 209 | 133 | . | 209 | 133 |

shows that this difference was due to the much higher proportion of cigarette smokers in the lung cancer group in Jersey.

The proportions of cigarette smokers in the control groups for the two islands were not significantly different ($P = 0{\cdot}5$) but in the lung cancer groups they differed by 16 % ($P = 0{\cdot}001$).

The lung cancer mortality rates of locally-born men smoking hand-rolled and manufactured cigarettes respectively on an age-adjusted basis are given in Table X.

TABLE X.—*Age-adjusted Lung Cancer Mortality Rates per* 100,000 *per Annum and Average Daily Levels of Cigarette Consumption of Locally Born Men Aged* 35+, *Smoking Cigarettes Only*

| | Lung cancer mortality rates per 100,000 per annum (Numbers of deaths in brackets) | | | | Average daily levels of cigarette consumption | | |
|---|---|---|---|---|---|---|---|
| | Hand-rolled cigarette smokers only | Manufactured cigarette smokers | All cigarette smokers | | Hand-rolled cigarette smokers only | Manufactured cigarette smokers | All cigarette smokers |
| Born in Jersey . . | 236 (14) | 269 (100) | 266 (114) | . | 27 | 27 | 27 |
| Born in Guernsey, Alderney or Sark . | 99 (14) | 184 ( 55) | 168 ( 69) | . | 20 | 31 | 29 |

The differences between the mortality rates of the two groups are greater than the differences between their average daily levels of cigarette consumption. The proportion of hand-rolled cigarette smokers in the control groups was significantly higher in Guernsey, Alderney and Sark than in Jersey ($P = 0{\cdot}005$ for locally-born plus U.K. immigrants; $P = 0{\cdot}04$ for locally born men only).

*Social class differences in lung cancer*

Before discussing the more important of the foregoing results, it is necessary to examine the differences between the various groups in their distribution among the Registrar-General's social classes, and to consider the possible effects of these differences on the lung cancer mortality rates. The U.K. immigrants came on the average from higher social classes than either the population of England and Wales generally, or the locally-born population in the Channel Islands. In no group, however, was there a significant difference between the social class distribution of the lung cancer and control groups.

Figures published by the Registrar-General of England and Wales (1958) have shown that there is a distinct social class gradient in male lung cancer mortality rates in England and Wales as these were greater in the lower than in the higher social classes. Thus, the standardized lung cancer mortality ratio for men aged 20–64 in England and Wales in 1949–53 ranged from 81 for Social

Class 1 to 118 for Social Class V. The average cigarette consumption per adult in the two classes in Britain in recent years (Todd, 1962), and probably for some time previously, was much the same. If British immigrants to Jersey had had the same social class distribution as locally-born men, and the same social class gradient of lung cancer as the U.K. population, the lung cancer mortality rate of the former would have been increased by just over 15 %.

### DISCUSSION

Four sets of comparisons are made in this paper and the discussion is simplified if each comparison is considered separately.

*British immigrants compared with locally-born men in Channel Islands*

British immigrants smoked slightly more cigarettes per head than locally-born men, but the differences were not significant (Table IV). On the other hand, the locally-born men may have smoked at the levels under which they were classified longer than the immigrants. The U.K.-born men had lower lung cancer rates than locally-born men in the Channel Islands in total and at all levels of cigarette smoking (Table VIII) but the differences were not significant. In total, the lung cancer mortality rates for locally-born men in the Channel Islands exceeded those of British immigrants during 1952–58 but were less than those of the immigrants during 1959–63. These differences were significant (Table VII). The British immigrants came, on the average, from higher social classes than locally-born men. Had the social class distribution of the two groups been comparable, the lung cancer mortality rate of U.K.-born cigarette smokers in Jersey would have increased by just over 15 % but it would still have been below the rate of locally-born men.

*Jersey compared with Guernsey, Alderney and Sark*

Locally-born men in Guernsey smoked slightly more than locally-born men in Jersey (Table IV) but the differences were not significant. On the other hand, the lung cancer rates in Guernsey, Alderney and Sark of locally-born men and British immigrants combined were significantly lower than those of their counterparts in Jersey (Tables V, VI). This suggests that there may have been a "Jersey factor" contributing to lung cancer in that island that did not occur, at least to the same extent, in Guernsey, Alderney and Sark. Table IX further suggests that the "Jersey factor" was to be found particularly among cigarette smokers.

*Residents of England and Wales compared with British immigrants to the Channel Islands*

British immigrants to the Channel Islands smoked more on the average than residents of Britain (Table IV). There was no significant difference between the lung cancer rate of male British immigrants to Jersey and that of men in England and Wales. If the former rate reflected the effects of a "Jersey factor", the latter may have reflected the effects of a "British factor". The male lung cancer rate of British immigrants to Guernsey, Alderney and Sark was significantly less than that of men in England and Wales (Table V). It is of course true that the British immigrants to the Channel Islands came from higher social classes on the average than residents of England and Wales, but this is unlikely to have accounted for all the difference between the male lung cancer rates of England and Wales and of British immigrants to Guernsey, Alderney and Sark. The U.K.-born men who came to live in Guernsey, Alderney and Sark thus provide another example, to set beside New Zealand, South Africa, Australia and U.S.A., of emigrants leaving Britain, smoking more in their new country of residence than men in Britain, and yet experiencing a lower lung cancer rate than the latter.

*Residents of England and Wales compared with locally-born men in the Channel Islands*

Locally-born men in the Channel Islands smoked more than residents of Britain (Table IV). On the other hand, the male lung cancer rate in England and Wales exceeded the locally-born rate in Guernsey, Alderney and Sark but was less than the rate in Jersey (Table V) ; neither difference was significant. The effect of the higher smoking rate in the Channel Islands therefore seems to have been offset by a factor in Britain that did not occur to the same extent in the islands.

What is the " British factor " to which the immigrants to the Channel Islands were no longer exposed ? While the climates of New Zealand, Australia, South Africa and U.S.A. all differ in important respects from that of Britain, this is not true of the Channel Islands. On the other hand, although more cigarettes per head were smoked in the Channel Islands than in England and Wales, the mortality rate from bronchitis in the Channel Islands was only 40 % of the rate in England and Wales. This suggests that air pollution in England and Wales may have contributed considerably to bronchitis, and it may also have been, at least in part, the " British factor " contributing to lung cancer to which immigrants to the Channel Islands were no longer exposed.

### CONCLUSIONS

Lung cancer is undoubtedly high in the Channel Islands. In Jersey there were 119 lung cancer deaths out of a total of 943 deaths (including some deaths of visitors) during the ten years 1952–61 among men aged 45–64. Thus, one in eight of all deaths among men aged 45–64 during this period was from lung cancer, and by 1961 this ratio had increased to one death in 5·4. In Guernsey, Alderney and Sark, there were 82 deaths from lung cancer out of a total of 597 deaths, including deaths of visitors, during 1952–61 among men aged 45–64. This was equal to approximately one death from lung cancer in every seven among men dying aged 45–64. In the 10 years 1952–61, only two male lung cancer deaths in Jersey and only four in Guernsey, Alderney and Sark occurred in non-smokers. On the other hand, 27 of the 65 women residents of the Channel Islands who died of lung cancer were non-smokers.

The main conclusions from the present survey are:

1. U.K.-born men who had become resident in the Channel Islands did not have a significantly different lung cancer mortality rate from locally-born men during the period 1952–61. It was found, however, that in the last few years, a trend of earlier years had been reversed and that the lung cancer mortality rate of British immigrants had exceeded that of locally-born men. British immigrants smoked slightly more cigarettes per head than locally born men in the Channel Islands but the differences were not significant.

2. The lung cancer mortality rate for locally-born men and British immigrants combined was higher in Jersey than in Guernsey, Alderney and Sark and the difference was significant. The differences between the two groups of Channel Islands may have been due to a factor particularly affecting Jersey. During the period 1952–61, there was an increase in the male lung cancer rates in both Jersey and Guernsey, Alderney and Sark. The increase was greater in Jersey than in the other three islands.

3. British-born men who settled in the Channel Islands smoked more cigarettes than men in England and Wales but did not have a significantly different lung cancer rate. When the two groups of Channel Islands were examined separately, it was found that the lung cancer rate of British immigrants to Guernsey, Alderney and Sark was significantly lower than that of men resident in England and Wales. The rate for men settling in Jersey was not significantly different from that of men in England and Wales.

4. The difference between the lung cancer mortality rate of locally-born men in the Channel Islands and of men in England and Wales was not significant, though the former smoked more than the latter.

Some of these conclusions correspond to conclusions reached in other enquiries. For example, in Guernsey, as in South Africa and U.S.A. British male immigrants smoked more than men in Britain and yet had lower lung cancer rates than the latter, pointing to the possible importance of exposure, earlier in life, of the immigrants to a " British factor " not found in the Channel Islands. This was not true of British male immigrants to Jersey perhaps because, as the comparison of lung cancer mortality rates in Jersey and Guernsey indicated, there was also a " Jersey factor ". In recent years, the highest lung cancer rate in the Channel Islands was found in British male immigrants and this raises the question whether the pattern of lung cancer mortality in the Channel Islands is coming into line with experience in New Zealand, South Africa, Australia and U.S.A., where British immigrants had higher lung cancer mortality rates than the locally-born men.

### APPENDIX

Passey (1962) has advanced the hypothesis that " the age when lung cancer develops is determined neither by the amount smoked nor the age at which smoking began." While I did not collect information about the age at which smoking began, the figures of those who died from lung cancer obtained in my second study in South Africa (Dean, 1962) and in the present study of the Channel Islands can be analysed by smoking habits and age at death. The South African figures showed that there was no significant gradient in the average age at death from lung cancer of non-smokers, light cigarette smokers, heavy cigarette smokers and other smokers, of rural and urban dwellers, or of U.K.-born and South African-born men, who died between the ages of 45 and 64.

The figures for the Channel Islands, where the analysis covered all ages, reveal a different picture. Considering Jersey-born men as an example, the average age at death from lung cancer declined from 72 for non-smokers and pipe or cigar smokers to 62 for those smoking cigarettes only. The differences between non-smokers, pipe or cigar smokers and mixed smokers are not significant for any of

TABLE XI.—*Average Age at Death of Male Lung Cancer Decedents*, 1952–61, *Jersey and Guernsey, Alderney and Sark*

| | Non-smokers | | Pipe or cigars only | | Mixed smokers | | Hand-rolled cigarettes only | | Cigarettes only (excluding hand-rolled only) | | Total | |
|---|---|---|---|---|---|---|---|---|---|---|---|---|
| | No. | Average age | No. | Average age | No. | Average age | No. | Average age | No. | Average age | No. | Average age |
| Jersey | | | | | | | | | | | | |
| Jersey-born men | 2 | 72 | 12 | 72 | 12 | 69 | 14 | 63 | 100 | 62 | 140 | 63 |
| U.K.-born men | — | — | 6 | 63 | 5 | 67 | 3 | 63 | 55 | 62 | 69 | 63 |
| Guernsey, Alderney and Sark | | | | | | | | | | | | |
| G.-, A.-, & S-born men | 4 | 61 | 14 | 65 | 20 | 62 | 14 | 65 | 55 | 58 | 107 | 60 |
| U.K.-born men | — | — | 4 | 72 | 3 | 73 | 1 | 54 | 18 | 63 | 26 | 65 |

Male smokers of cigarettes only (excluding smokers of hand-rolled cigarettes only).

| | 1–10 daily | | 11–22 daily | | 23+ | |
|---|---|---|---|---|---|---|
| | No. | Average age | No. | Average age | No. | Average age |
| Jersey—Locally and U.K.-born combined | 17 | 66 | 51 | 62 | 87 | 61 |
| G., A. and S.—Locally and U.K.-born combined | 3 | 63 | 23 | 59 | 47 | 58 |

the groups in Table XI, and the only significant difference between the age at death of handrolled and manufactured cigarette smokers is in Guernsey-, Alderney- and Sark-born men ($P < 0{\cdot}01$). The difference between non-cigarette and cigarette smoking groups is however significant ($P < 0{\cdot}05$) in all but U.K.-born Jersey men, and this group was not significantly different from the others. When the cigarette smokers are classified by the amount smoked, the numbers in each group are small, but the downward trend in age at death was significant ($P = 0{\cdot}05$) in Jersey though not in Guernsey where there were only 3 in the 1–10 cigarettes per day group.

### REFERENCES

Darling, A. S.—(1958–61) *Reports of the Public Health Committee.*
Dean, G.—(1961) *Brit. Med. J.*, ii, 1599.—(1962) *Ibid.*, ii, 120.
Goodman, L. A.—(1963) *J. Roy. Stat. Soc.*, A, **126**, 94.
Mork, T —(1962) *Acta med. scand.*, suppl. 384.
Passey, R. D.—(1962) *Lancet*, ii, 107.
Todd, G. F.—(1962) Private communication and *Statistics of Smoking in the UK.*, Research Paper No. 1, Tobacco Research Council, London.
Registrar General of England and Wales.—(1958) Decennial Supplement, 1951. Occupational Mortality, Part II, Vol. I Commentary. London (H.M. Stationery Office).